# Pre-trauma cognitive traits predict fear generalization and associated prefrontal functioning in a longitudinal rodent model

**DOI:** 10.1038/s41386-025-02263-4

**Published:** 2025-11-04

**Authors:** László Szente, Manó Aliczki, Gyula Y. Balla, Róbert D. Maróthy, Zoltán K. Varga, Bendegúz Á. Varga, Zsolt Borhegyi, László Biró, Kornél Demeter, Christina Miskolczi, Zoltán Balogh, Huba Szebik, Anett Szilvásy-Szabó, Anita Kurilla, Máté Tóth, Éva Mikics

**Affiliations:** 1 Translational Behavioural Neuroscience Research Group, HUN-REN Institute of Experimental Medicine, Budapest, Hungary; 2 Doctoral School, Semmelweis University, Budapest, Hungary; 3 Behavioural Studies Unit, HUN-REN Institute of Experimental Medicine, Budapest, Hungary; 4 Integrative Neuroendocrinology Research Group, HUN-REN Institute of Experimental Medicine, Budapest, Hungary; 5 Institute of Enzymology, HUN-REN Research Centre for Natural Sciences, Budapest, Hungary

**Keywords:** Stress and resilience, Post-traumatic stress disorder

## Abstract

Posttraumatic stress disorder (PTSD) is a chronic psychiatric condition that develops in susceptible individuals exposed to traumatic stress. Identifying predisposing risk factors and mechanisms presents a significant challenge for prevention and therapy development. Here, we aimed to identify behavioral predictors of excessive fear generalization - a core symptom of PTSD - and its neural correlates in rats using a longitudinal design. Prior to trauma, rats underwent extensive behavioral test batteries to assess their emotional and cognitive traits. They were then exposed to a single traumatic experience via inescapable footshocks. Twenty-eight days later, fear generalization was measured in a neutral/safe context, differentiating vulnerable (high freezing) and resilient (low freezing) subpopulations. Reduced pre-trauma operant learning performance emerged as the strongest predictor of excessive fear generalization. Neuronal activity mapping revealed altered medial prefrontal cortex (mPFC) dynamics in vulnerable animals, characterized by activity changes and less coordinated activity-coupling between subregions. Gene expression analysis and cell-specific activity labeling pointed to VIP/CRH+ interneurons as potential mediators of excessive fear. As a molecular intervention, silencing prefrontal *Crh* expression after fear memory consolidation markedly enhanced mPFC activation and reduced fear expression, supporting a regulatory role of this interneuron population in fear processing. As a behavioral intervention, post-trauma operant training facilitated the reduction of generalized fear and led to changes in plasticity-related gene expression in the mPFC, indicating overlapping neural circuits governing operant learning and fear regulation. These findings together highlight operant learning and mPFC network functions as vulnerability markers and mediators of excessive fear generalization, with implications for prevention and targeted therapy in PTSD.

## Introduction

Adaptive fear responses and preparatory anxiety states are essential for survival, by initiating or priming adaptive behavioral responses to cope with danger. However, improper threat detection and inappropriate initiation of defensive behaviors that are inadequate for the context are hallmarks for maladaptive conditions such as anxiety disorders and posttraumatic stress disorder (PTSD)[1]. PTSD is a chronic psychiatric condition that develops following exposure to overwhelming stressors, characterized by complex symptomatology including intrusive memories, avoidance, hyperarousal, and cognitive and mood alterations. However, a central feature of PTSD is generalized fear expressed in safe contexts—a core symptom also observed in several anxiety disorders, where anxiety emerges in the absence of actual threat. Notably, only 10–20% of individuals develop chronic PTSD symptoms, underscoring the importance of pre-existing vulnerability factors in disease etiology [2, 3]. Identifying pre-trauma risk factors is critical for improving prevention strategies, particularly in high-risk populations, and for informing targeted interventions [4].

Traditionally classified as an “Anxiety disorder”, but now categorized as “Trauma- and stressor-related disorder” [1, 5], PTSD is increasingly understood through its cognitive dimensions, particularly the persistence of intrusive, generalized, and extinction-resistant fear memories [6–8], alongside broader cognitive impairments [9]. Neuropsychological studies consistently show PTSD-related deficits in verbal memory, executive function, and contextual processing, which predict symptom persistence and poor treatment outcomes [10]. These cognitive impairments are also current therapeutic targets, though interventions like exposure therapy still require refinement [11]. While human studies often cannot distinguish between pre-trauma alterations and those induced by trauma exposure, landmark twin and prospective studies indicate that pre-trauma cognitive abilities contribute to PTSD vulnerability [12–16]. Mechanistically, PTSD has been linked to altered prefrontal-hippocampal engagement during fear learning and extinction, suggesting that pre-existing differences in these circuits may influence vulnerability, though causal relationships remain unclear [17, 18].

A detailed understanding of cellular and circuit-level alterations underlying PTSD, particularly in relation to fear generalization, is essential for developing more effective prevention and treatment [19, 20]. Given the difficulty of assessing premorbid traits in humans, longitudinal animal models are critical to complement clinical PTSD research [21, 22]. To improve cross-species translation, complex disorders like PTSD [1] must be deconstructed into biologically relevant domains, as proposed in the Research Domain Criteria (RDoC) framework [23]. RDoC-based approaches allow modeling of specific predisposing factors and symptom outcomes, offering more precise insights into underlying mechanisms and translationally relevant targets [22, 24]. Accordingly, we focused on contextual fear generalization, a core, evolutionarily conserved feature of PTSD, thereby maximizing the translational validity of our findings [25, 26].

In this study, we used a longitudinal rat model of trauma-induced fear generalization to: (1) identify pre-trauma traits within cognitive and negative valence domains that predict excessive fear generalization following trauma; (2) characterize prefrontal cortical mechanisms of vulnerability; and (3) test whether cognitive training mitigates generalization via prefrontal circuit engagement. We show that pre-trauma deficits in operant learning predict heightened fear generalization, associated with medial prefrontal cortex (mPFC) dysfunction involving CRH+ interneurons. Post-trauma cognitive training enhanced the extinction of generalized fear and facilitated circuit plasticity, offering a translational framework linking cognitive vulnerability and neural circuitry in PTSD.

## Methods

Comprehensive details of all methods can be found in the Supplementary Material.

### Animals

Adult male Long-Evans rats (Charles River) were group housed (4/cage) under controlled conditions (22 ± 1 °C, 50 ± 10% humidity, reverse 12-h light/dark cycle). Experiments were carried out in accordance with the Directive of the European Parliament and the Council (2010/63/EU) and approved by the Hungarian Government Office (PE/EA/874-5/2020) and the local Animal Welfare Committee.

### Trauma exposure and fear generalization assessment: identification of resilient and vulnerable subpopulations

**Fig. 1:**
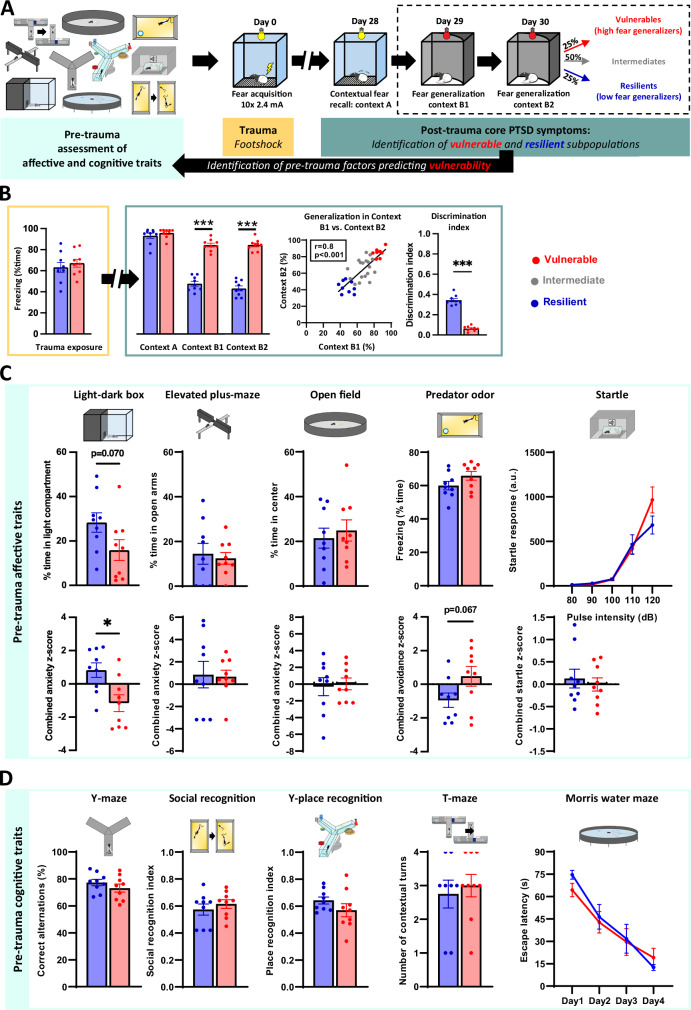
Evaluation of the predictive power of pre-trauma affective and cognitive traits for trauma-induced fear generalization in Experiment #1. **A** Experimental design illustrating a wide behavioral battery before trauma exposure (footshock in CtxA), and subsequent long-term (28 days) testing of contextual fear recall (CtxA), and generalized fear in a safe context (CtxB) on two consecutive days. Subjects were categorized into resilient and vulnerable groups as upper and lower quartiles based on their average freezing levels in CtxB1 and 2. **B** Resilient and vulnerable groups exhibited distinct fear responses in CtxB1-2 (generalized fear), despite similar responses during acquisition (*p* = 0.525) and contextual fear recall in CtxA (*p* = 0.386). Individual fear responses in CtxB were stable (trait-like) across testing days indicated by a strong positive correlation. Context discrimination index (CtxA − CtxB)/(CtxA + CtxB) used as another measure of fear generalization, differed significantly between resilient and vulnerable groups. **C** Affective pre-trauma traits in vulnerable and resilient groups. Differences in anxiety-like behaviors were observed only under heightened threat conditions. Specifically, vulnerable groups showed increased avoidance of aversive zones in the Light-dark box (%time in light: *p* = 0.070; *z*-score: *p* = 0.010) and Predator odor test (freezing: *p* = 0.125; *z*-score: *p* = 0.067). No differences were found in the less aversive Elevated Plus-Maze (%time in open arm: *p* = 0.708; *z*-score: *p* = 0.900) and Open Field (%time in center: *p* = 0.606; *z*-score: *p* = 0.816) tests. Similarly, no differences were observed between groups in acoustic startle responses (*p* = 0.408). **D** Cognitive pre-trauma traits in vulnerable and resilient groups. Vulnerable and resilient groups showed no difference in working memory performance in the Y-maze (*p* = 0.293), social recognition (*p* = 0.289), in contextual memory in the Y-place recognition test (*p* = 0.202), in their contextual-habitual strategies in the T-Maze (*p* = 0.730), and spatial learning in the Morris Water Maze (*p* = 0.751). All data are shown as mean ± SEM, *p < 0.05, **p < 0.01, ***p < 0.001 using one-way ANOVA, repeated measures ANOVA, Mann–Whitney test, and Pearson correlation.

Subjects underwent a 7-min session of 10 electric footshocks (2.4 mA, 30 s inter-trial intervals). Contextual fear was assessed 28 days later in the original context (CtxA) for 5 min. On the subsequent two days, fear generalization was assessed in a novel context (CtxB: all visual, olfactory, and tactile components of the context altered) for 20 min (Fig. 1A), except in Experiment #4 where we applied 10-min CtxB session to avoid extinction before operant training. Fear proxy variable was time spent with freezing, a typical fear response in rodents, measured by Ethovision XT15 software (Noldus, The Netherlands). Cohorts (*n* ≥ 32) were stratified into vulnerable and resilient subpopulations (upper and lower quartiles) based on average freezing responses in CtxB.

### Behavioral testing

Tests were conducted during the early dark phase. Tests were video recorded and analyzed using Ethovision XT15 software, except in operant chambers or startle system, where behavioral data were collected from automated apparatus.

### Anxiety tests: light-dark box (LD), elevated plus-maze (EPM), and open field (OF)

Anxiety was assessed by measuring time spent, and the number and latency of entries in the brightly illuminated zone of the LD, in the open arms of the EPM, and the center of the OF. We also combined time%, entries and latencies into aversive zones to composite *z*-scores for a more reliable index of anxiety.

### Startle response assessment

Startle reactivity was measured in SR-LAB Startle Response System (San Diego Instruments). Startle stimuli between 80 and 120 dB intensities were presented as before [27, 28].

### Predator odor avoidance test

Innate fear response was assessed by exposing subjects to a synthetic predator odor component, 2-methyl-2-thiazoline (2MT; M83406, Sigma Aldrich) presented in the corner of a Plexiglas arena for 10 min.We assessed approaches to 2MT source and freezing as indexes of innate fear.

### Y-maze

Spontaneous alternation (turns into novel arms) as the index of working memory was assessed in an arena consisting of three interconnected Plexiglas arms during a 5 min period.

### Social recognition test

The procedure, adapted from Engelmann [29], assessed social recognition by measuring the time spent with social investigation of an unfamiliar juvenile rat vs. a familiar juvenile.

### Y-place context recognition test

A transparent Y-shaped apparatus was contextualized with objects along the walls. Subjects explored the start arm and an additional choice arm for 10 min, then were reintroduced to both choice arms 30 min later. Time spent in the novel arm indicated place recognition.

### Morris water maze

The water maze was a 180 cm circular water-filled pool, with visual cues around. A submerged platform was placed in one quadrant. Subjects were placed at random release points for four daily trials (max 90 s) over four days. After reaching the platform, subjects stayed for 15 s before the next trial. Average escape latency was the learning performance index.

### T-maze

The apparatus had three interconnected arms, with two choice arms distinguished by tactile and visual cues at the entry zones. After habituation, subjects were trained to choose a cornflake-rewarded arm. During test phase, the start arm was reversed, where habitual or contextual response strategy was assessed.

### Simple operant and Go/No-Go tasks

Tasks were conducted in automated operant chambers (MedAssociates, USA) with nose-poke holes, infrared sensors, LED lights, and a sucrose pellet receptacle (BioServ, USA). Subjects underwent two operant learning phases with daily sessions, where a light cue signaled the rewarded hole for 30 s/10 s in phases 1 and 2, respectively. Phase completion criterion was >80% correct response accuracy. In the subsequent Go/No-Go task, subjects had to withhold responses during a 5 s No-Go acoustic cue co-presented with the light cue in 50% of trials. Total responses, accuracy, premature responses, and omissions were measured.

### Complex operant task, strategy set-shifting task, and 5-choice serial reaction time test (5-CSRTT)

The same operant boxes were equipped with five nose-poke holes for a complex task where the location of a hole indicated the reward, while the light cue was irrelevant. After reaching 80% accuracy, subjects switched to a strategy set-shifting task where the light cue predicted the reward. They then progressed to 5-CSRTT based on [30], requiring pokes during cue periods that gradually shortened.

### Post-trauma operant training

One week after the fear generalization assessment in CtxB, vulnerable and resilient subpopulations were sorted into “trained” and “yoked control” groups. “Trained” groups performed the complex operant task (see above), while “yoked controls” collected rewards randomly, matching trained group medians. After 4 weeks of training, fear generalization and its extinction in CtxB were re-tested.

### Gene expression analysis

mPFC and hippocampal samples were collected immediately after fear generalization in CtxB and qPCR was performed using custom-made TaqMan Array Cards measuring 45 or 95 candidate gene expression (Applied Biosystems, USA). Expression was normalized to *Actb* and *Gapdh* and relative quantity was calculated by the 2-ddct method [31].

### Immunohistochemistry

90 min after CtxB exposure, subjects were anesthetized and transcardially perfused. On 30 µm coronal sections, c-Fos as activity marker was labeled with cell-type markers (somatostatin (SST), parvalbumin (PV), calretinin (CR), vasoactive intestinal peptide(VIP)) and monoaminergic fibers (serotonin transporter(SERT), tyrosine hydroxylase(TH), dopamine beta hydroxylase(DBH)) using multiple fluorescent immunolabeling as reported [32]. For details, including antibodies, see Supplementary Materials.

### Microscopy and image analysis

Slides were imaged with a digital slide scanner (3DHISTECH, Hungary). Section planes and anatomical structures were defined based on the reference atlas [33]. C-Fos signals were counted in ImageJ, with manual co-localization (VIP, CR, PV, SST) across 2–3 sections bilaterally (for details of regions of interest and quantification, see Supplementary Materials). TH, DBH, and SERT in the PrL were imaged via Nikon C2 Confocal Microscope (z-stack) and processed in ImageJ for fiber density quantification.

### *Crh* knockdown using small hairpin RNA (shRNA) vectors

AAV5 vectors expressing shRNA targeting rat *Crh* mRNA or a scrambled sequence (Fig.S6) were bilaterally injected into the mPFC two days post-trauma. Virus infections were confirmed by EGFP immunolabeling.

### Statistical analysis

Data are presented as mean ± standard error of the mean. Statistical analysis involved Student’s *t* test, ANOVA with Tukey’s post hoc, repeated measures ANOVA, and Mann–Whitney U tests where appropriate. Significance was set at *p* < 0.05. Feature importance was assessed using Random Forest Classifiers.

## Results

### Unpredictable footshock induces lasting fear memory with persisting fear generalization in the vulnerable subpopulation: the validity of the model

Since our goal was to identify pre-trauma trait-like characteristics that determine vulnerability to trauma, we first needed to confirm that our stratification method reliably and validly distinguishes vulnerable and resilient subpopulations (i.e., high vs. low fear generalizers) without major confounding factors (Experiment #1). In our experimental paradigm, rats underwent a single session of unpredictable footshocks, followed by a 4-week undisturbed period to model the progressive-persistent nature of PTSD. Then fear responses were assessed in both trauma-associated (CtxA, to confirm contextual fear learning) and safe contexts (CtxB, to assess fear generalization as core symptom of PTSD) (Fig. 1A). Footshock exposure resulted in robust and lasting contextual fear memory with minimal population variance (Fig. 1B). In contrast, fear generalization in CtxB exhibited high variability, with a marked difference between upper and lower quartiles, defining vulnerable and resilient groups (Fig. 1B). This difference was stable across testing days (Fig. 1B), confirming that fear generalization is indeed an individual trait and do not originate from contextual fear learning or memory recall performance. Context discrimination index showed also significant difference (Fig. 1B). Noteworthy, we did not observe cagemate effect in the distribution of vulnerable-intermediate-resilient groups, suggesting that long-term fear generalization is not affected by social contagion or buffering in as shown in other paradigms (Khi-square: χ² = 74.06, df = 87, *p* = 0.84).

A random forest analysis of behavioral data (footshock, CtxA, CtxB) identified average freezing in CtxB as the key feature distinguishing vulnerable and resilient subpopulations (Supplementary Fig. S2A, B), which was used throughout the study. Separation of subpopulations was further supported by our meta-analysis of all study cohorts (for details, see Supplementary Table S3) by showing a bimodal tendency in fear generalization in CtxB (Supplementary Fig. S2E**)** as unimodal distribution was rejected (Excess mass = 0.077, *p* < 0.001), although the presence of two modes could not be shown (Excess mass = 0.028, *p* = 0.3). Such trends were not present during fear acquisition and contextual fear recall in CtxA (Supplementary Fig. S2C, D). Subpopulations also differed in their within-session reduction of fear in CtxB, indicating diminished safety-learning in vulnerable subjects (Supplementary Fig. S2F). In summary, stable vulnerable, and resilient subpopulations can be defined enabling us to investigate individual pre-trauma traits predictive for subsequent fear generalization.

### Pre-trauma affective traits are weak predictors of fear generalization

Having confirmed the validity of our stratification approach (Fig.1B), we next retrospectively analyzed pre-trauma behavioral traits of our experimental subgroups in Experiment #1 to determine which traits predicted trauma-induced fear generalization (for detailed pre-trauma experimental design, see Supplementary Fig. S1). We evaluated pre-trauma affective traits, given PTSD’s historical classification as an anxiety disorder [1, 5]. A retrospective analysis of pre-trauma anxiety traits did not reveal significant differences between groups in the EPM and the OF (Fig. 1C). However, the vulnerable group exhibited higher avoidance tendencies in more aversive conditions, such as the LD and predator odor test (Fig. 1C). No differences were observed in pre-trauma acoustic startle reactivity. These findings suggest that pre-trauma anxiety-like behaviors under heightened threats have limited predictive capacity for fear generalization following trauma.

### Pre-trauma cognitive vulnerability factors: operant learning and cognitive flexibility predict fear generalization

While lower cognitive performance has been linked to PTSD, its role in PTSD vulnerability and associated neurobiological changes remains unclear [9, 10, 14, 15]. To address this, we also conducted a series of pre-trauma cognitive assessments to evaluate their predictive power for post-trauma fear generalization.

**Fig. 2:**
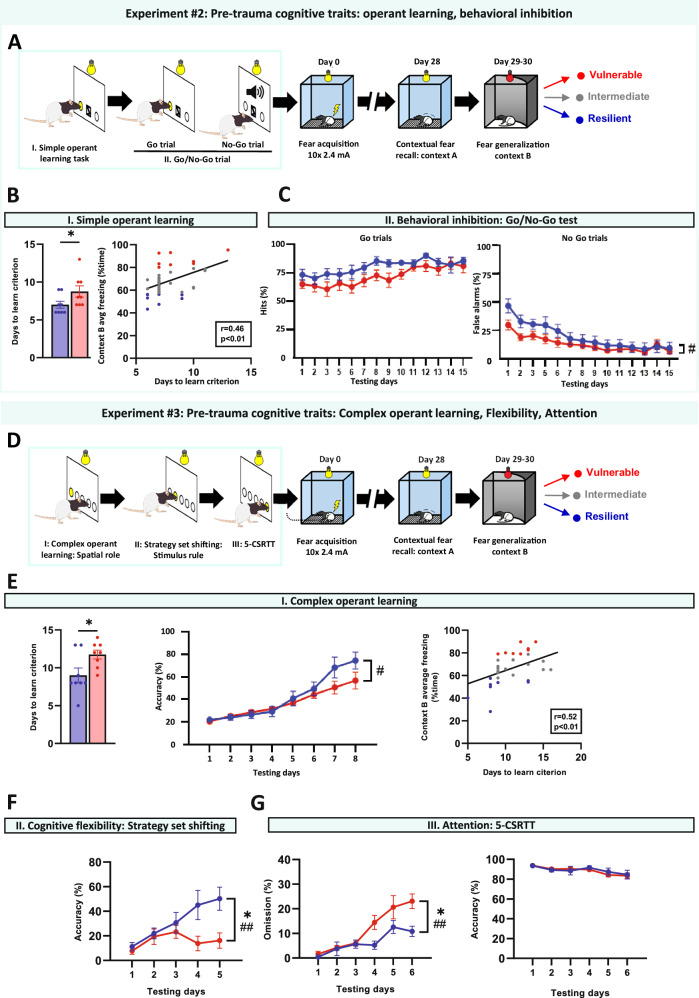
Impaired pre-trauma operant learning and cognitive flexibility predict enhanced post-trauma fear generalization. **A** Schematic experimental design for pre-trauma assessment of operant learning (simple operant task) and behavioral inhibition (Go/No-Go task) in Experiment #2. **B** Operant learning in vulnerable and resilient groups. Vulnerable subjects exhibiting high fear generalization required significantly more days to learn a simple operant task (defined as >80% accuracy across 2 consecutive days) compared to resilient subjects (*p* = 0.040) (left panel). Operant learning performance negatively correlated with fear generalization across the entire population (including intermediate quartiles; right panel). **C** Behavioral inhibition in vulnerable and resilient groups. The Go/No-Go task revealed a significant group*day interaction for false alarms (NoGo errors, *p* = 0.017), with vulnerable subjects demonstrating fewer errors during the initial phase of testing. Go response rates showed trend-like differences between groups (*p* = 0.058), suggesting that vulnerable subjects exhibited generally decreased responding across all stimuli rather than specific deficits in inhibitory control. **D** Experimental design for pre-trauma assessment of operant learning (complex operant task), cognitive flexibility (set-shifting), and attention (5-Choice Serial Reaction Time Task: 5-CSRTT) in Experiment #3. **E** Similarly to the simple operant task, vulnerable subjects needed more days to learn a more complex operant task compared to the resilient subjects (left panel), which was also reflected in lower response accuracy (days to learn: *p* = 0.028, accuracy: *p* = 0.032; interaction: *p* = 0.009; middle panel). Performance on the complex operant task negatively correlated with fear generalization across the entire population (right panel). **F** The Strategy Set-Shifting task also indicated cognitive inflexibility in the vulnerable group indicated by slower increase in accuracy (*p* = 0.017). **G** In the 5-CSRTT, vulnerable subjects maintained comparable accuracy (*p* = 0.483; right panel) but exhibited higher omission rates than resilient subjects (*p* = 0.040; interaction effect: *p* = 0.009; left panel). All data are presented as mean ± SEM. *p < 0.05, **p < 0.01, ***p < 0.001, significant difference from the resilient group; #*p* < 0.05, ##*p* < 0.01, significant interaction; Unpaired t-test, Mann–Whitney U test, One-way ANOVA with Tukey’s post hoc test, or Repeated measures ANOVA as appropriate.

Analyses revealed no significant between-group differences in pre-trauma working memory, social or place recognition, spatial learning, or habitual-contextual strategies (Fig. 1D). In contrast, vulnerable subjects demonstrated significantly impaired pre-trauma operant learning, requiring more days to master an operant task compared to resilient subjects (Experiment #2: Fig. 2A, B). Pre-trauma operant performance negatively correlated with fear generalization across the entire population, including intermediate quartiles (Fig. 2B). We confirmed this finding in a separate cohort using a more complex operant learning paradigm (Experiment #3: Fig. 2D, E).

The inhibitory function assessed via the Go/No-Go task revealed a significant day*group interaction for No-Go errors (Fig. 2C, right panel), while a trend in Go responses (Fig. 2C, left panel) indicated generally reduced responding in vulnerable subjects, not selective inhibitory deficits.

Vulnerable subjects also displayed reduced cognitive flexibility in the set-shifting task (Fig. 2F) and increased omissions in the 5-CSRTT with intact accuracy (Fig. 2G), suggesting attention deficits or generally inhibited responding. In summary, pre-trauma deficits in operant learning performance, and cognitive flexibility emerged as the strongest predictors of post-trauma fear generalization.

### Post-trauma cognitive training facilitates reduction of generalized fear through plasticity-related gene expression changes in prefrontal and hippocampal regions

**Fig. 3:**
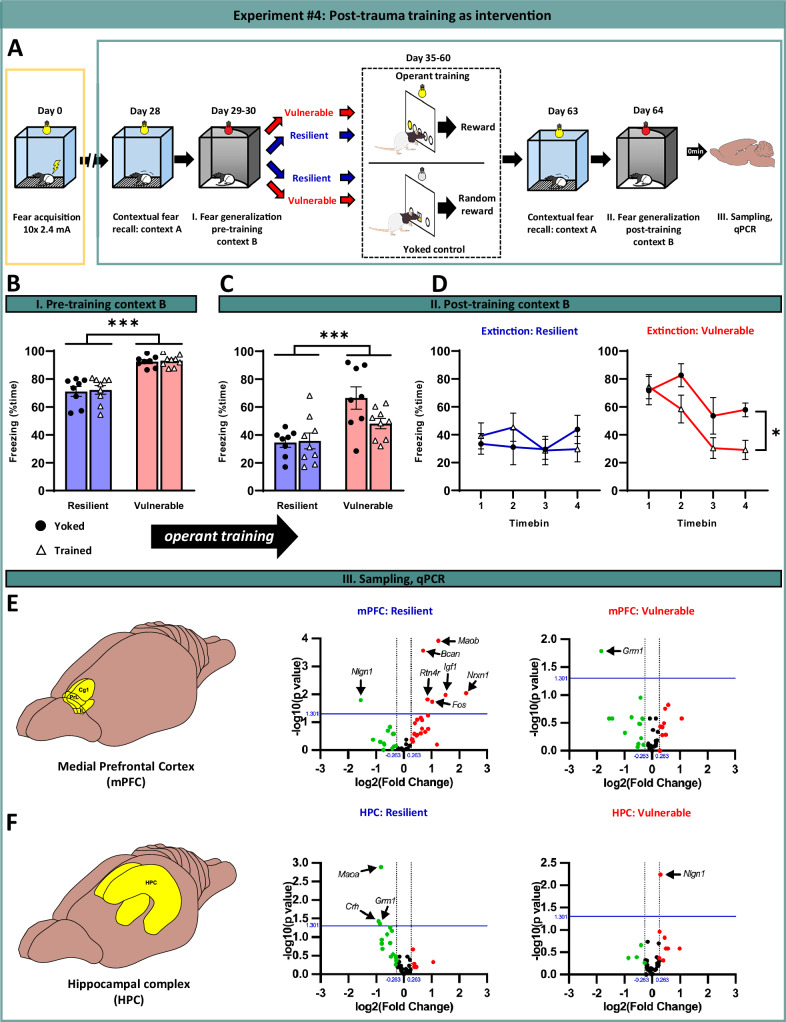
Post-trauma operant training facilitates the reduction of generalized fear in vulnerable subjects. **A** Schematic representation of the design of Experiment #4 with trauma exposure, brief fear recall testing in traumatic (CtxA, 5 min) and safe (CtxB, 10 min) contexts before operant training (complex task as used in Experiment #3), that was followed again by fear recall testing in CtxA and B. **B** Pre-training generalized fear responses were carefully counterbalanced between trained and yoked control subgroups within both resilient and vulnerable subpopulations (p > 0.720). **C** Following operant training intervention, generalized fear responses remained significantly elevated in the vulnerable group compared to resilient counterparts. **D** Vulnerable subjects that received operant training exhibited significantly accelerated reduction of generalized fear compared to yoked subjects (*p* = 0.047), indicating a training-specific therapeutic effect. This was not apparent in the resilient group (*p* = 0.859), likely due to a floor effect in this population **E** Volcano plots depicting differentially expressed genes in the medial prefrontal cortex (mPFC) and **F** hippocampus (HPC) following operant training intervention relative to yoked controls in both vulnerable and resilient groups. Genes meeting significance thresholds (*p* < 0.05, indicated by blue horizontal line) with biologically relevant expression changes (>1.2-fold, indicated by vertical dashed lines) are highlighted. Significantly upregulated genes are denoted in red and downregulated genes in green. Key plasticity-related genes identified including, *Bcan* (brevican), *Igf1 (*insulin-like growth factor 1), *Nlgn1 (*Neuroligin 1), *Nrxn1 (*Neurexin 1), *Rtn4r* (Nogo receptor), *Grm1* (metabotropic glutamate receptor 1), besides others such as *Fos*, *Crh* (corticotropin releasing hormone), *Maoa and Maob (*monoamine oxidase A and B). All data are presented as mean ± SEM. *p < 0.05, ***p < 0.001; Two-way ANOVA with Tukey’s post hoc test, or repeated measures ANOVA.

Operant learning performance strongly predicted fear generalization, suggesting shared neural mechanisms between cognition and fear regulation. Therefore, we investigated whether targeted engagement of these shared circuits through post-trauma operant training could serve as a ‘therapeutic intervention’ to mitigate fear generalization and enhance learning of context safety (Experiment #4: Fig. 3A). We used the complex operant task as training paradigm as a cognitively challenging task that recruits prefrontal networks potentially involved in both operant learning and fear expression. Vulnerable and resilient subjects were assigned to operant training intervention or yoked control conditions, balanced for pre-training freezing in CtxB (Fig. 3B). Groups also showed similar freezing during trauma and pre-training CtxA exposure (Supplementary Fig. S3A, B). Contextual fear remained intact post-training, with only a slight decrease in resilient animals (Supplementary Fig. S3C). Crucially, operant training accelerated reduction of generalized fear in vulnerable subjects (Fig. 3D, right panel).

To investigate the molecular basis of this beneficial effect of cognitive training, we analyzed expression of 45 genes implicated in PTSD vulnerability -identified through human GWAS and candidate gene studies —including neurotransmitter receptors, modulators, plasticity-related molecules (e.g., SERT, CRH, and glucocorticoid receptor, brain-derived neurotrophic factor [34–36]). In addition, we analyzed genes representing fundamental network functions such as excitation-inhibition balance, stress signaling, and activity markers (for details, see Supplementary Table S1). Analyses focused on the mPFC and hippocampus—regions critically involved in both PTSD pathophysiology and learning processes (6, 8), sampled immediately after post-training CtxB testing. As shown in Fig. 3E, F and Supplementary Table S1, trained versus yoked controls displayed distinct gene expression patterns across resilient and vulnerable groups. Our exploratory molecular analysis revealed that operant training induced significant transcriptional changes in genes related to synaptic plasticity in both groups: *Bcan-*brevican, *Grm1*-metabotropic glutamate receptor 1, *Igf1-*insulin-like growth factor 1, *Nlgn1*-neuroligin1, *Nrxn1-*Neurexin 1, *Rtn4r*-Nogo receptor, besides neuronal activity (*Fos*), interneuron marker (*Crh*), and monoaminergic signaling (*Maoa* and *Maob*) (Fig. 3E, F).

### Differential activation patterns in the fear network of vulnerable and resilient subpopulations

**Fig. 4:**
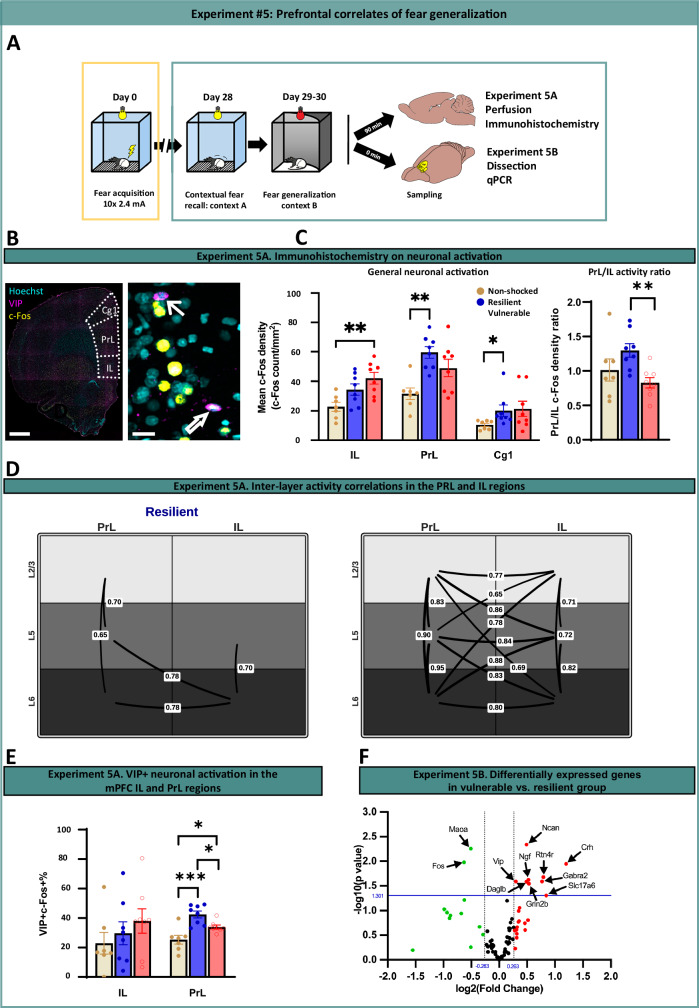
Prefrontal mechanisms of high fear generalization. **A** Schematic illustration of the experimental design of Experiment #5A and 5B. **B** Representative photomicrographs depicting immunohistochemical labeling of vasoactive intestinal polypeptide (VIP, purple), and c-Fos (yellow) in the mPFC. Filled arrows indicate VIP+ neurons, while empty arrows denote VIP/c-Fos double-labeled neurons. Scale bars: 1 mm (left), 20 µm (right). **C** Prefrontal subregions were differentially activated in vulnerable and resilient groups indicated by c-Fos neuronal activation marker in Experiment #5A. The infralimbic cortex (IL) showed significant activation exclusively in the vulnerable group (*p*(shock)=0.006; post hoc vulnerable vs. control *p* = 0.004, resilient vs. control *p* = 0.082), whereas the prelimbic cortex (PrL) and anterior cingulate cortex (Cg1) exhibited significant activation selectively in the resilient group (PrL: *p*(shock)=0.004; post hoc vulnerable vs. control *p* = 0.085, resilient vs. control *p* = 0.003; Cg1: *p*(shock)=0.030; post hoc vulnerable vs. control *p* = 0.186, resilient vs. control *p* = 0.029). This was reflected in significant change in PrL/IL activity ratio between vulnerable and resilient groups (right panel). **D** Correlation matrix of L2/3, L5, and L6 layers of the PrL and IL cortices. Whereas all layers/subregions exhibited significant positive correlation in the vulnerable group (15 inter-regional correlations; *r* = 0.65–0.95), only 5 significant inter-regional correlations (*r* = 0.65–0.78) emerged in the resilient group. Line thickness represent the significance of correlation coefficients (numbers on lines). **E** Quantification of VIP + neuronal activation in the PrL, demonstrating significantly enhanced activity in the resilient group (*p*(shock) < 0.001; post hoc vulnerable vs. resilient: *p* = 0.032). **F** Volcano plot illustrating differentially expressed genes in the mPFC of vulnerable versus resilient subjects in Experiment #5B. Significantly upregulated genes are highlighted in red and downregulated genes in green (*p* < 0.05 (uncorrected) indicated by blue horizontal line; fold-change threshold of 1.2 indicated by vertical dashed lines). Gene abbreviations and p levels: *Crh*: Corticotropin-releasing hormone (*p* = 0.011); *Daglb*: Diacylglycerol lipase beta (*p* = 0.026); *Fos*: Fos proto-oncogene (*p* = 0.010); *Gabra2*: GABA-A receptor, alpha 2 subunit (*p* = 0.025); *Grin2b*: NMDA receptor 2B subunit (*p* = 0.028); *Maoa*: Monoamine oxidase A (*p* = 0.005); *Ncan*: neurocan (*p* = 0.004); *Ngf*: Nerve growth factor (*p* = 0.023); *Rtn4r*: Nogo receptor (*p* < 0.001); *Slc17a6*: Vesicular glutamate transporter 2 (*p* = 0.049); *Vip*: Vasoactive intestinal peptide (*p* = 0.025).

To identify elements of the fear circuitry [19, 20] exhibiting functional changes related to enhanced fear generalization, we immunolabeled c-Fos activity marker following CtxB exposure in Experiment #5 (Fig. 4A–C and Supplementary Fig. S4). No significant activation differences were found across multiple hippocampal subregions, the paraventricular thalamus, or basolateral amygdala. However, the central amygdala showed reduced activation in vulnerable compared to resilient subjects (Supplementary Fig. S4A). The most significant effects appeared in the mPFC, with distinct subregion-specific patterns and shifts (Fig. 4C). Shock/trauma evoked significant activation in a sub-region dependent manner: infralimbic (IL) and prelimbic (PrL) cortices were significantly activated in the vulnerable and resilient subpopulations, respectively. Accordingly, there was a significant shift in PrL/IL activation ratio between vulnerable and resilient populations (Fig. 4C, right panel). Similar activity patterns were observed when we analyzed PrL and IL in a quasi-layer-specific manner (Supplementary Fig. S4B, C) [37–39]. Interestingly, the correlation analysis of PrL and IL layers revealed further functional differences between vulnerable and resilient groups. Whereas all layers of PrL and IL exhibited positive correlation in the vulnerable group (non-regulated activity coupling, i.e., 15 inter-regional correlations), only highly selective couplings (i.e., 5 inter-regional correlations, including PrL and IL deep-layers) emerged in the resilient group (Fig. 4D), suggesting fine-tuned network function in the resilient group. Importantly, PrL-IL connections were implicated in the regulation of fear extinction and new learning [40, 41]. The anterior cingulate cortex (Cg1) showed a PrL-like activation pattern, though less pronounced. Given the critical involvement of the mPFC in both operant learning processes and fear regulation [42], and the observed significant and differential activation changes in mPFC subregions of vulnerable versus resilient subjects, we conducted further investigation into network-level and molecular mechanisms underlying vulnerability.

### Characterization of mPFC network function reveals altered interneuron activity and monoaminergic inputs in vulnerable group

Next, we assessed activity changes in specific interneuron populations of the mPFC due to their critical role in orchestrating network activity. Immunohistochemical analysis revealed that prelimbic VIP+ interneurons were activated during CtxB after prior shock exposure, but they exhibited significantly lower activation in vulnerable (Fig. 4E), which aligned with the observed reduction in global PrL activity (Fig. 4C). While c-Fos activity of VIP+ interneurons in the IL also reflected the directional trend of global c-Fos activity differences, this difference did not reach statistical significance (Fig. 4E). In contrast to the marked differences observed in VIP+ interneurons, other interneuron subtypes showed no significant difference in activation levels between groups, including PV-, CR-, and SST-positive interneurons (Supplementary Fig. S5). These findings underscore the selective impact of VIP+ interneuron dysfunction on local network activity, which may underlie the elevated fear generalization in vulnerable subjects.

Given the decreased *Maoa* gene expression in vulnerable subjects and the first line application of monoaminergic drugs in PTSD and fear-related disorders [43], we also investigated monoaminergic innervation of the mPFC (Supplementary Fig. S6). While individual markers for dopaminergic (TH), noradrenergic (DBH), and serotonergic (SERT) inputs showed no group differences, combined catecholaminergic density (TH + DBH) indicated significantly lower input in the vulnerable group, implying altered catecholaminergic signaling in high fear generalizer subjects (Supplementary Fig. S6B–E).

### Differential gene expression profiles in the mPFC associated with fear generalization

We conducted a comprehensive gene expression analysis in the mPFC of 92 candidate genes previously implicated in PTSD pathogenesis and fundamental neuronal network functions [34–36], encompassing genes regulating monoaminergic, glutamatergic, GABAergic, cannabinoid, neuropeptide neurotransmission, neuronal plasticity, HPA-axis function, and inflammation. This gene set was an extension of the 45 genes investigated following operant training, allowing for a more comprehensive analysis while essentially covering the same functional categories and candidate gene groups (for the complete list, see Supplementary Table S2). As shown in Fig. 4F, vulnerable subjects showed significant downregulation of *Fos* and *Maoa*, suggesting reduced neuronal activity and monoamine degradation compared to resilient counterparts. In contrast, vulnerable animals exhibited upregulation of multiple plasticity-related genes (*Ncan*, *Rtn4r*, *Ngf*), interneuron markers (*Crh*, *Vip*, *Gabra2*), and additional neurotransmission-related genes (*Grin2b*, *Slc17a6*, *Daglb*), indicating broad alterations in synaptic signaling. Full expression data are provided in Supplementary Table S2.

### Prefrontal *Crh* knockdown induces neuronal hyperactivity in association with reduced fear expression

**Fig. 5:**
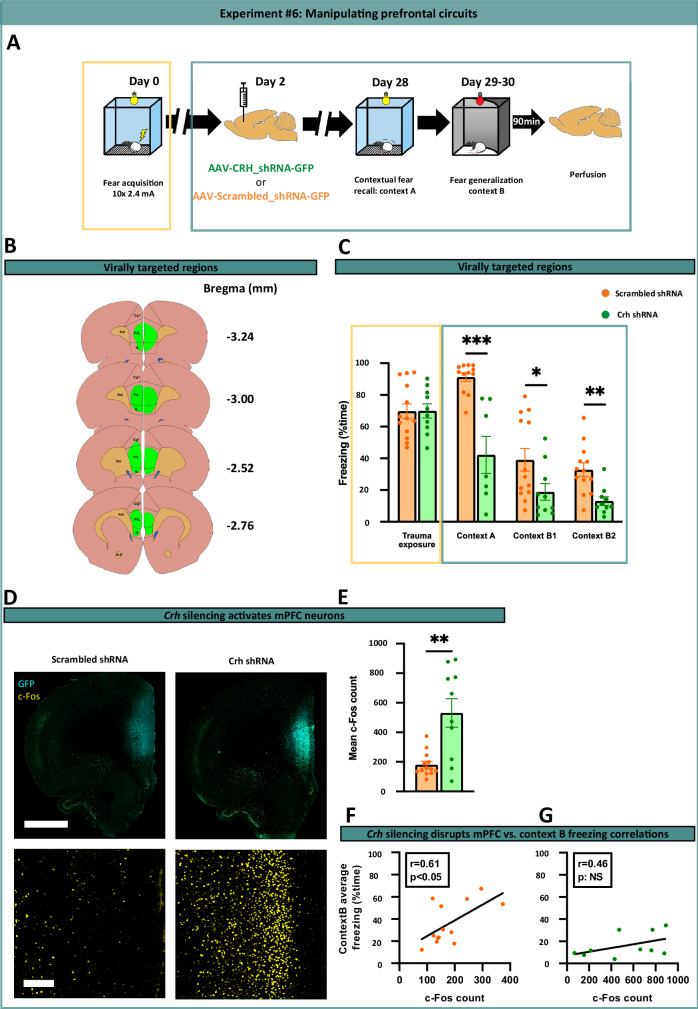
Prefrontal *Crh* knockdown induces neuronal hyperactivity in association with reduced fear expression. **A** Experimental design of targeted prefrontal knockdown of *Crh* gene expression in Experiment #6. To avoid interference with fear acquisition and memory consolidation, viral vector administration was performed two days after trauma exposure. **B** Illustration of virus injection sites in mPFC. **C** *Crh* shRNA and scrambled RNA (control) groups were counterbalanced for their freezing response exhibited during trauma exposure (*p* = 0.975). *Crh* knockdown induced significant reductions in fear expression across multiple contexts, including the trauma-associated (CtxA: *p* < 0.001) and safe contexts (CtxB1 and B2: *p* = 0.027; *p* = 0.001, respectively). **D**, **E** *Crh* shRNA markedly enhanced c-Fos activity in the mPFC (*p* = 0.006). **F** Correlation analysis revealing a significant positive relationship between prefrontal neuronal activity and generalized fear in controls. **G** The correlation between prefrontal neuronal activity and generalized fear was abolished following *Crh* knockdown. Data presented as mean ± SEM *p < 0.05, **p < 0.01, ***p < 0.001. One-way ANOVA with Tukey’s post hoc test, unpaired t-test, Mann–Whitney U test, or Spearman correlation.

The elevated expression of prefrontal *Crh* and *Vip mRNA*, coupled with differential activation patterns in VIP+ interneuron populations observed in vulnerable subjects, implied a potential causal role for these cell types and signaling pathways in fear generalization vulnerability. Given the significant co-expression of these neuropeptides (50–80%) in cortical interneurons [44, 45], the significant change in VIP activity, and the well-documented impact of enhanced CRH neurotransmission on stress-related behaviors [28, 45–48], we targeted this cell population to test their causal involvement in fear generalization through gene silencing. We used shRNA to knock down *Crh* in the mPFC post-trauma (Fig. 5, Supplementary Fig. S7). Four weeks later, *Crh* knockdown induced significant reductions in fear expression across multiple contexts, including the trauma-associated and safe contexts (Fig. 5C), and increased mPFC activation (Fig. 5D, E). Unlike scrambled controls, *Crh* knocked-down animals showed no correlation between mPFC activity and freezing (Fig. 5F, G), indicating altered network function and related behavioral output. These results demonstrate that disruption of CRH signaling in prefrontal interneurons exerts profound effects on prefrontal network output and related functions, including fear expression across contextual boundaries. These findings support the critical involvement of medial prefrontal circuits in modulating fear expression and generalization, and point to CRH signaling and CRH+ interneuron populations as key mediators of these circuits and their associated behavioral outputs.

## Discussion

Our study demonstrates the significant contribution of specific pre-trauma cognitive traits to the development of trauma-induced fear generalization in a longitudinal rat model. We identified lower operant learning performance and reduced cognitive flexibility as key predictors of excessive fear generalization. Furthermore, post-trauma operant training facilitated the learning of context safety, i.e., the reduction of generalized fear in vulnerable subjects, suggesting overlapping neural substrates for operant and fear learning processes. Neurobiological analyses revealed that the mPFC serves as a critical mediator of these effects through altered CRH/VIP positive interneuron activity, and associated gene expression changes. Targeted knockdown of *Crh* expression markedly reduced fear expression, underscoring the functional role of these interneurons in fear generalization.

To address the need for longitudinal PTSD models capable of dissecting pre-trauma vulnerability factors, we investigated trauma-induced contextual fear generalization in rats, preceded by detailed cognitive-affective profiling. Meta-analysis of our paradigm confirmed that inescapable footshock evoked persistent fear responses in the traumatic context across the study population, and variable levels of fear generalization in a novel context that was stable across tests as an individual trait. The observed variability in fear generalization could not be attributed to differences in fear acquisition or contextual recall, ruling out potential confounds in fear learning performance. Our approach of identifying vulnerable and resilient subpopulations based on fear generalization quartiles is supported by human PTSD prevalence rates [2, 3], previous rodent studies [49–51], and comprehensive statistical analyses (distribution analyses and machine learning classification) [52, 53]. This methodological framework provides a robust platform for investigating pre-trauma vulnerability factors of pathological fear generalization, a well-established and quantifiable phenotype associated with anxiety disorders and PTSD.

While human studies have documented cognitive deficits in PTSD patients [12, 54], that determine symptoms trajectory and severity [16, 54–56], the causal relationship between these impairments and PTSD development remains unclear [54]. Our findings provide compelling evidence that specific cognitive deficits precede and predict vulnerability to trauma-induced fear generalization. Specifically, we demonstrated that lower pre-trauma operant learning capacity and reduced cognitive flexibility consistently predicts excessive post-trauma fear generalization. Noteworthy, we replicated this effect in an independent cohort tested under different pre-trauma conditions (thereby excluding potential confounding effects of pre-trauma test types and sequence), and using a more demanding operant task, thereby confirming the robustness of this behavioral predictor.

Other cognitive domains showed weaker associations: striatal-hippocampal network dependent habitual memory rigidity (T-maze) [57, 58], did not correlate with vulnerability, highlighting the specificity of prefrontal-dependent cognitive functions in this process [57, 59]. Similarly, working memory and social/contextual and spatial learning abilities were not predictive of fear generalization, despite their previously proposed contributions to PTSD [6, 60, 61]. These differential associations indicate that operant learning selectively engages neural circuits underlying trauma-induced fear generalization, aligning with the specific focus of our model on generalization rather than the broader spectrum of PTSD symptoms typically assessed in human studies [62].

Our model confirmed that negative emotionality and anxiety traits as predispose to PTSD-like symptoms [13, 63], but only under high-threat conditions. Notably, affective traits showed weaker predictive power than cognitive factors. We hypothesize that anxiety may more strongly influence other PTSD symptoms, such as avoidance and hyperarousal, not assessed here. This aligns with the RDoC framework’s emphasis on domain-specific investigations of psychiatric disorders [23]. Our results suggest that different cognitive-affective traits may differentially contribute to specific PTSD symptom clusters, with operant learning and cognitive flexibility particularly relevant to intrusive symptoms and fear generalization.

Our results have significant implications for clinical interventions. A major clinical objective is to modulate neurocircuits underlying vulnerability through pharmacological interventions, behavioral therapies, or their combination to alleviate symptoms. While extinction enhancers have been widely studied [11, 64], fewer studies explored the potential benefits of cognitive training, such as playing the Tetris game to interfere with memory consolidation of fearful or traumatic stimuli [65–68]. The few studies examining cognitive training’s impact on PTSD-related neurobiological deficits [69], despite limited statistical power, have indicated promising effects. Our study corroborates these observations, confirming that operant training positively influences subsequent reduction of generalized fear by enhanced safety learning.

Gene expression analysis following operant training revealed alterations primarily in plasticity-related genes, previously implicated in PTSD, including metabotropic glutamate receptor 1 [70] and neuroligin 1 [71, 72]. These data support the hypothesis that cognitive training recruits neural circuits involved in both operant and fear learning, fostering plasticity-related adaptations that enhance network operation, context differentiation, and modulate fear expression. The observed transcriptional changes in both groups indicate that training markedly affected plasticity-related pathways, although with limited explanatory power for specific mechanisms. Given the established role of mPFC in both operant learning/cognitive flexibility and fear regulation [42, 73, 74], our finding positions this region as a critical mediator of the observed cross-talk between these functions.

Examination of fear network activity [75, 76], revealed differential activation patterns in prefrontal subregions between vulnerable and resilient subjects during fear generalization, corresponding with volumetric and functional deficits reported in human PTSD [8, 18]. Noteworthy, we detected modest trauma-induced c-Fos activation across regions during long-term assessment, i.e., the second safe context exposure one month after trauma, with significant activation detected solely in the mPFC. Beyond the overall activation, there was a significant proportional shift between mPFC subregions, i.e., prelimbic and infralimbic, suggesting that their interconnectivity or coordinated action on downstream regions might underlie the differences in generalized fear between resilient and vulnerable groups. We also propose that more subtle local circuit differences could mediate vulnerability. Our global c-Fos counts showed that high fear generalizers exhibited lower activity in PrL but higher activity in IL. This contrasts with previous findings where PrL drives fear expression and IL activity promotes fear extinction/inhibition [77–79], assuming higher activity equals greater efficacy. However, considering the limitations of c-Fos labeling, such as insensitivity to temporal dynamics or finer functional characteristics, higher activity might also reflect desynchronized or ineffective network. Our correlation analyses support this hypothesis by showing non-selective co-activation of PrL-IL layers in vulnerable subjects, whereas resilient subjects exhibited highly selective co-activation/coupling, including deeper layer interconnectivity. Latter finding corresponds with previous studies showing this connection as a crucial regulator of fear expression and extinction besides new learning [40, 41, 80, 81]. Taken together—its relevance to operant learning as a primary behavioral predictor of fear generalization, the magnitude of shock-induced activity changes, and the functional significance of IL/PrL activity balance in fear regulation—the mPFC offered strong justification for our subsequent in-depth investigation.

Further investigation of mPFC network characteristics revealed significant alterations in VIP+ interneurons in vulnerable subjects, evidenced by changes in c-Fos activity and *Vip* mRNA expression in addition to *Crh* levels. Since VIP+ interneurons play essential roles in network synchronization and regional output regulation [73, 82, 83], their altered function can significantly shape network dynamics and adaptive behavioral responses such as fear learning and expression [84–86]. Stress effects can affect prefrontal networks via VIP+ neurons, considering their significant overlap with CRH+ interneurons, which are highly stress-sensitive and mediate stress effects [28, 46, 47]. Indeed, silencing *Crh* expression in mPFC induced marked prefrontal hyperactivation (likely reflecting the network orchestrating function of these disinhibitory interneurons [83]) and significantly reduced fear expression. Moreover, this hyperactivation of mPFC resulted in the loss of correlation between neuronal activation and freezing behavior following *Crh* knockdown. While the exact mechanisms remain to be clarified, our findings indicate that intact coordination of prefrontal networks by VIP/CRH+ interneurons is essential for generating proper network outputs to shape adaptive fear responses. A key direction for future research will be to determine whether and how CRH signaling within this circuitry mediates the predictive relationship between pre-trauma cognitive traits and post-trauma fear generalization.

In summary, our comprehensive investigation of neurobehavioral vulnerability traits in trauma-related fear generalization highlights PFC-dependent cognitive function as a key determinant of vulnerability. We identified a strong association between lower pre-trauma operant performance and excessive fear generalization, which could be mitigated by post-trauma operant training. The degree of vulnerability was reflected in prefrontal functions across multiple levels, including altered subregional activity, gene expression profiles related to plasticity, and specific interneuron functioning. These findings suggest that prefrontal-dependent cognitive traits may serve as valuable neurobehavioral markers for predicting fear generalization in PTSD. Furthermore, they provide a neurobiological foundation for developing targeted preventive measures and cognitive training interventions to alleviate maladaptive fear generalization, favoring resilience. Future studies should explore the translational potential of these findings in clinical populations.

## Supplementary information

Supplementary Material

## Data Availability

The datasets generated during and/or analyzed during the current study are available from the corresponding author on reasonable request.
