# Supplementary material for: Pre-trauma cognitive traits predict fear generalization and associated prefrontal functioning in a longitudinal rodent model
Source: Neuropsychopharmacology. 2025 Nov 4;51(5):881–94. doi: 10.1038/s41386-025-02263-4 (PMC13013975; doi:10.1038/s41386-025-02263-4)
Supplement: Supplementary file 1 — Supplementary Material [file 41386_2025_2263_MOESM1_ESM.docx]

**Supplementary Fig. S1. Experimental designs and timelines for all experiments**. **A** Experiment #1 testing the predictive power of affective and cognitive pre-trauma traits for trauma-induced fear generalization. **B** Experiment #2 testing the predictive power of pre-trauma operant learning performance and behavioral inhibition (Go/NoGo task) for trauma-induced fear generalization**. C** Experiment #3 testing the predictive power of pre-trauma operant learning performance (more complex task), cognitive flexibility (Set-shifting), and attention (5-CSRTT) for trauma-induced fear generalization**. D** Experiment #4 testing operant training (complex) as a post-trauma therapeutic intervention to reduce fear generalization and enhance safety learning. **E** Experiment #5A and 5B investigating the prefrontal correlates of vulnerability vs. resilience, i.e. low and high fear generalization. Perfused samples from Experiment #5A were used to immunolabel c-Fos with cell type-specific markers, whereas microdissected samples from Experiment #5B were used for qPCR analysis of differentially expressed candidate genes. **F** Experiment #6 tested the impact of prefrontal *Crh* silencing on fear expression and generalization.

**Supplementary Fig. S2. Machine-learning based freezing behavior analysis reveals distinct vulnerability phenotypes following trauma exposure**. **A-B** Feature importance analyses, derived from the average of 30 independent calculations, identified CtxB average freezing as the most robust predictor for distinguishing between the animal phenotypes. This finding is consistent across the optimized Random Forest classification algorithm (A) and our established group definition methodology (B). Tests were divided into equal-length timebins (Shock1-Shock3 indicate equal timebins during fear acquisition, whereas CtxB/1-CtxB/8 indicate 5min consecutive timebins during CtxB1 and CtxB2 testing; CtxA was not further divided) and average freezing levels were also calculated. The results demonstrate strong congruence in other discriminating factors, highlighting the freezing phenotype in the altered context as the primary factor that differentiates the groups. **C-E** Meta-analysis of standardized freezing behavior (z-scores) across multiple experimental cohorts using identical trauma exposure and fear recall testing protocols (for cohorts and details, see Supplementary Table S3). Histograms display freezing z-score distributions across the complete population sample (including intermediate phenotypes), overlaid by density estimations (thick grey lines). On the x-axes, individual z-scores are presented as rug plots and colored by freezing phenotypes (resilient in blue, vulnerable in red, intermediate subjects are omitted for visual clarity). **C** Freezing during trauma (footshock/fear acquisition), and **D** Freezing during CtxA exposure exhibited normal distributions with no significant differences between vulnerable and resilient groups as subjects were interspersed along the x-axis. **E** Freezing in CtxB demonstrated a separation of vulnerable and resilient groups by definition. Moreover, density estimation shows that distribution is not unimodal (unimodal distribution rejected by Excess mass=0.077, p<0.001), although solid bimodality could not be statistically confirmed. **F** Freezing time curves in CtxB point out impaired ‘extinction’ (reduction of generalized fear) in vulnerable animals indicated by a significant time*group interaction besides different freezing levels. All data presented as mean values ± S.E.M.; Statistical significance determined by two-way repeated measures ANOVA with Tukey's post hoc test; ***p<0.001 for group effect; #p<0.05 for group-time interaction.

**Supplementary Fig. S3. Fear recall dynamics during trauma exposure and Context A before and after operant training. A** Vulnerable group exhibited significantly elevated freezing behavior during trauma exposure compared to the resilient group, although the effect size was modest. **B** The freezing response was similar between groups during pre-training exposure to CtxA. **C** Following operant training, freezing responses in CtxA remained elevated across all groups, with a slight reduction observed in the resilient group (p=0.001). Notably, trained and yoked groups exhibited similar fear responses across testing conditions: trauma exposure (p=0.367), and both pre-training (p=0.692) and post-training CtxA exposures (p=0.798), suggesting that differences in fear generalization in CtxB (see Fig. 4) were not attributable to the fear acquisition or contextual recall differences. All data are presented as mean ± S.E.M. Statistical significance was determined using two-way ANOVA with Tukey’s post hoc test for multiple comparisons. *p<0.05, **p<0.01.

**Supplementary Fig. S4. Neuronal activity mapping during fear generalization. A** C-Fos activity exhibited during Context B exposure revealed similar activation patterns across most examined regions between vulnerable and resilient phenotypes. The medial prefrontal cortex (see Fig. 5) and the central amygdala (CeA) emerged as notable exceptions, latter exhibiting significantly reduced neuronal activity in vulnerable subjects compared to resilient counterparts (p=0.025; Tukey’s post hoc p=0.024). Quantitative analysis included key nodes within the extended fear circuitry: basolateral amygdala (BLA; p=0.444), CeA, paraventricular thalamic nucleus (PVT, p=0.242), dorsal and ventral hippocampal subfields (dCA1-3, vCA1-3), and dentate gyrus regions (dDG, vDG) (dorsal: CA1: p=0.166; CA3: p=0.753; dentate gyrus (DG): p=0.31; ventral: CA1: p=0.299; CA3: p=0.844; DG: p=0.084)). **B-C** A quasi-layer-specific (L2/3, L5, L6) mapping of the medial prefrontal cortex revealed significantly increased prelimbic (PrL) and infralimbic (IL) activation in the resilient and vulnerable groups, respectively, similar to overall activity changes of these regions (Fig.5). All data are presented as mean ± S.E.M. Statistical significance was determined using two-way ANOVA with Tukey’s post hoc test for multiple comparisons. *p<0.05.

**Supplementary Fig. S5. Interneuron subtype-specific activity in the medial prefrontal cortex during fear generalization testing (ContextB exposure).** The left panels show representative photomicrographs demonstrating immunofluorescent labeling of c-Fos with specific interneuron markers (empty arrows), alongside their co-localization (filled arrows). Right panels provide quantative analysis of activation within distinct interneuron populations across mPFC subregions. The examination revealed no significant differences between groups in the activation patterns of **A** parvalbumin-positive (PV+) interneurons, **B** calretinin-positive (CR+) interneurons, or **C** somatostatin-positive (SOM+) interneurons within either the infralimbic (IL) or prelimbic (PrL) cortices. Scale bar: 50 µm. All data are presented as mean ± S.E.M. Statistical analysis was performed using one-way ANOVA.

**
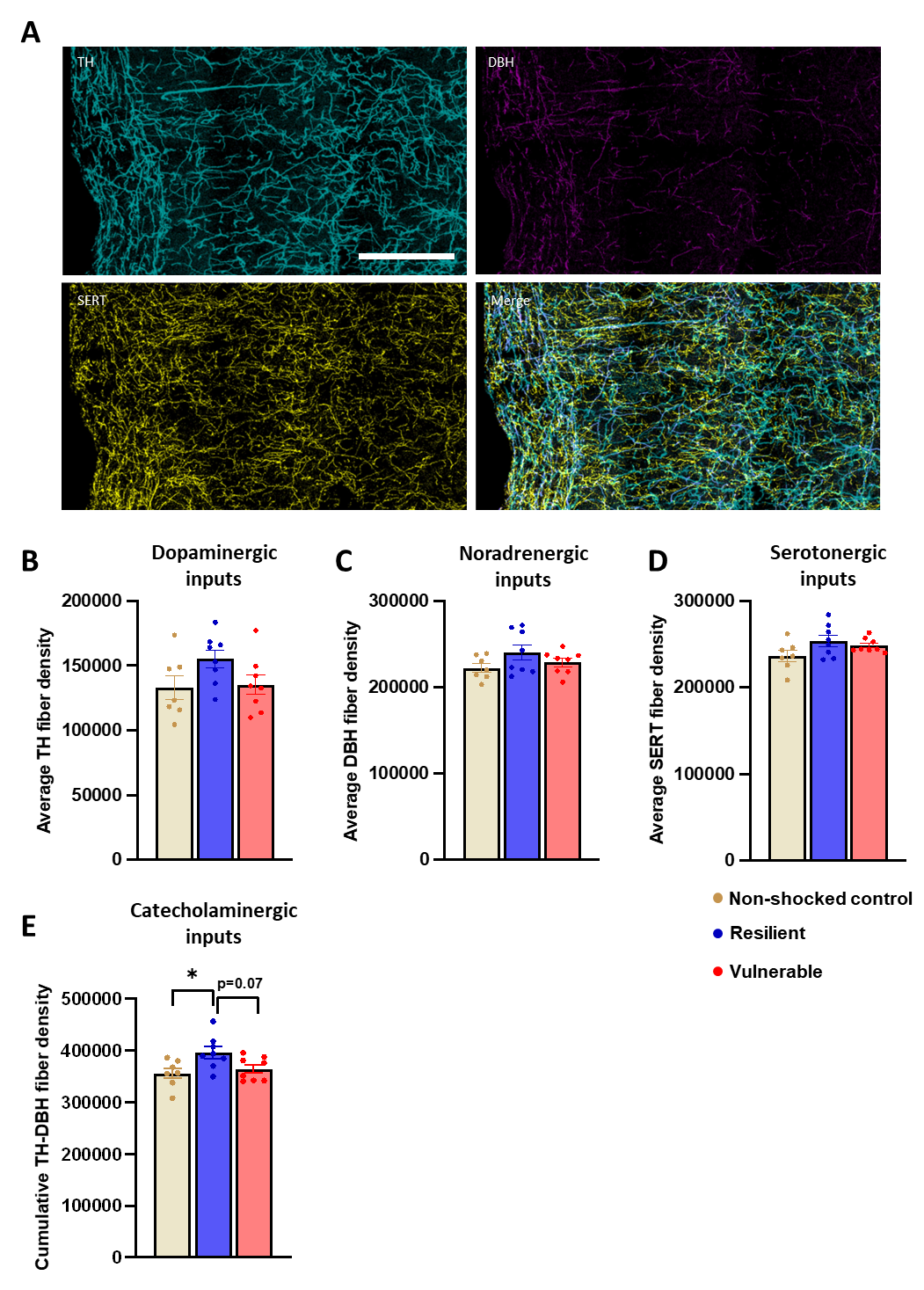
**

**Supplementary Fig. S6. Quantitative analysis of monoaminergic innervation in the mPFC during fear generalization testing (ContextB exposure). (A)** Representative photomicrographs demonstrating triple immunofluorescent labeling of monoaminergic fiber markers within the prelimbic cortex. Individual channel analysis revealed no statistically significant differences between groups in the density of **B** tyrosine hydroxylase-positive (TH+) fibers (dopaminergic innervation), **C** dopamine beta-hydroxylase-positive (DBH+) fibers (noradrenergic innervation), or **D** serotonin transporter-positive (SERT+) fibers (serotonergic innervation). However, **E** analysis of cumulative catecholaminergic innervation density (combined TH+ and DBH+ fiber quantification) demonstrated significantly elevated values in resilient subjects. Scale bar: 500 µm. All data are presented as mean ± S.E.M. Statistical analysis was performed using one-way ANOVA.

**Supplementary Fig. S7. Validation of AAV-shRNA-mediated Crh knockdown in the medial prefrontal cortex. A-B** The ‘cnc’ construct (red underline) showed the strongest efficacy in reducing *Crh* expression with non-significant trend at 2 weeks (p=0.081), and significant reduction after 4 weeks (**p<0.01; B). Constructs ‘rbk’ and ‘bpn’ induced no significant reduction in *Crh* levels at tested titers and with the current statistical power (n=3 mice/group). Accordingly, ‘cnc’ construct with 4-week incubation period was selected for behavioral experiments. All data are presented as mean ± S.E.M. **p<0.01. Statistical analysis was performed using one-way ANOVA with Tukey’s post hoc test for multiple comparisons.

**Table S1. Gene expression changes in resilient and vulnerable groups following operant training compared to yoked control subgroups.**

| **Medial prefrontal cortex** | | | | | | |
| --- | --- | --- | --- | --- | --- | --- |
| **Resilient** | | |  | **Vulnerable** | | |
| **Gene** | **p value** | **Fold change** |  | **Gene** | **p value** | **Fold change** |
| **Maob** | **0.0001** | **2.367** |  | **Grm1** | **0.016** | **0.279** |
| **Bcan** | **0.0003** | **1.622** |  | Grin1 | 0.111 | 0.746 |
| **Nrxn1** | **0.009** | **4.736** |  | Sst | 0.150 | 1.499 |
| **Igf1** | **0.010** | **2.834** |  | Ngf | 0.176 | 1.389 |
| **Rtn4r** | **0.015** | **1.808** |  | Vip | 0.250 | 0.595 |
| **Nlgn1** | **0.016** | **0.340** |  | Gabra2 | 0.262 | 2.095 |
| **Fos** | **0.018** | **2.037** |  | Nrxn1 | 0.262 | 0.947 |
| Grin2a | 0.057 | 1.832 |  | Bdnf | 0.262 | 0.368 |
| Ncan | 0.069 | 1.527 |  | Nfkb2 | 0.262 | 0.339 |
| Calb1 | 0.081 | 1.395 |  | Drd1 | 0.266 | 0.789 |
| Grin1 | 0.083 | 1.578 |  | Rtn4r | 0.319 | 1.348 |
| Crhr1 | 0.109 | 1.319 |  | Gria2 | 0.327 | 0.720 |
| Gabra2 | 0.148 | 0.709 |  | Fos | 0.331 | 0.739 |
| Maoa | 0.168 | 1.572 |  | Calb1 | 0.367 | 1.233 |
| Fosb | 0.170 | 1.841 |  | Igf1 | 0.423 | 1.026 |
| Pvalb | 0.200 | 0.671 |  | Bcan | 0.457 | 1.156 |
| Acan | 0.221 | 1.711 |  | Grin2b | 0.475 | 0.507 |
| Npas4 | 0.252 | 1.528 |  | Npy | 0.515 | 1.410 |
| Gria1 | 0.262 | 1.302 |  | Daglb | 0.522 | 1.288 |
| Cck | 0.262 | 0.779 |  | Acan | 0.592 | 0.759 |
| Daglb | 0.262 | 0.762 |  | Grin2a | 0.649 | 1.131 |
| Ncam1 | 0.285 | 1.393 |  | Gria1 | 0.667 | 1.095 |
| Gad1 | 0.298 | 1.376 |  | Crhr1 | 0.673 | 1.120 |
| Vip | 0.412 | 1.245 |  | Npas4 | 0.707 | 1.107 |
| Gad2 | 0.423 | 1.220 |  | Maob | 0.743 | 1.104 |
| Gabra1 | 0.423 | 1.061 |  | Arc | 0.745 | 0.908 |
| Grin2b | 0.423 | 0.463 |  | Calb2 | 0.749 | 0.706 |
| Igf2 | 0.489 | 1.256 |  | Gad1 | 0.779 | 1.092 |
| Crh | 0.503 | 0.555 |  | Crh | 0.779 | 0.804 |
| Npy | 0.588 | 0.609 |  | Gad2 | 0.802 | 0.960 |
| Nfkb2 | 0.631 | 2.287 |  | Gabra1 | 0.803 | 0.907 |
| Calb2 | 0.631 | 0.616 |  | Fosb | 0.815 | 1.100 |
| Arc | 0.692 | 1.160 |  | Cck | 0.839 | 1.043 |
| Bdnf | 0.711 | 0.821 |  | Nlgn1 | 0.844 | 1.053 |
| Drd1 | 0.873 | 0.947 |  | Maoa | 0.855 | 0.694 |
| Sst | 0.873 | 0.865 |  | Ncan | 0.861 | 0.958 |
| Ngf | 0.873 | 0.748 |  | Dlg4 | 0.912 | 0.968 |
| Grm1 | 0.924 | 1.058 |  | Igf2 | 0.914 | 0.937 |
| Dlg4 | 1.000 | 0.980 |  | Pvalb | 0.999 | 1.000 |
| Gria2 | 1.000 | 0.601 |  | Ncam1 | 1.000 | 1.216 |
| Ntrk1 | N/A | N/A |  | Ntrk1 | N/A | N/A |
| Ntrk2 | N/A | N/A |  | Ntrk2 | N/A | N/A |
| Oxtr | N/A | N/A |  | Oxtr | N/A | N/A |

| **Hippocampus** | | | | | | |
| --- | --- | --- | --- | --- | --- | --- |
| **Resilient** | | |  | **Vulnerable** | | |
| **Gene** | **p value** | **Fold change** |  | **Gene** | **p value** | **Fold change** |
| **Maoa** | **0.001** | **0.564** |  | **Nlgn1** | **0.006** | **1.226** |
| **Crh** | **0.037** | **0.529** |  | Gad2 | 0.109 | 1.208 |
| **Grm1** | **0.043** | **0.551** |  | Bdnf | 0.150 | 1.362 |
| Grin1 | 0.055 | 0.706 |  | Ngf | 0.200 | 1.178 |
| Ngf | 0.067 | 0.739 |  | Dlg4 | 0.220 | 0.755 |
| Maob | 0.084 | 0.657 |  | Gabra1 | 0.262 | 1.974 |
| Ntrk1 | 0.116 | 0.574 |  | Grm1 | 0.262 | 1.482 |
| Gria2 | 0.145 | 0.707 |  | Npy | 0.262 | 1.406 |
| Nrxn1 | 0.150 | 0.572 |  | Fos | 0.409 | 0.681 |
| Igf2 | 0.206 | 0.583 |  | Fosb | 0.423 | 0.553 |
| Arc | 0.214 | 1.247 |  | Gria2 | 0.429 | 1.202 |
| Ncam1 | 0.288 | 0.760 |  | Ncam1 | 0.479 | 0.848 |
| Cck | 0.324 | 0.819 |  | Ntrk1 | 0.482 | 1.318 |
| Nfkb2 | 0.337 | 0.721 |  | Arc | 0.490 | 0.878 |
| Bcan | 0.360 | 0.818 |  | Vip | 0.508 | 1.188 |
| Pvalb | 0.405 | 1.158 |  | Nfkb2 | 0.508 | 0.853 |
| Gabra1 | 0.423 | 0.826 |  | Bcan | 0.546 | 0.830 |
| Ntrk2 | 0.438 | 0.825 |  | Igf1 | 0.562 | 1.127 |
| Calb2 | 0.462 | 0.885 |  | Acan | 0.562 | 0.862 |
| Npy | 0.465 | 2.085 |  | Calb2 | 0.573 | 1.172 |
| Sst | 0.522 | 1.292 |  | Sst | 0.581 | 1.142 |
| Ncan | 0.525 | 0.849 |  | Crh | 0.613 | 0.887 |
| Daglb | 0.549 | 0.816 |  | Oxtr | 0.631 | 1.176 |
| Drd1 | 0.584 | 1.137 |  | Calb1 | 0.637 | 1.135 |
| Gria1 | 0.593 | 0.871 |  | Grin2a | 0.670 | 0.881 |
| Gabra2 | 0.594 | 0.931 |  | Rtn4r | 0.696 | 0.878 |
| Fosb | 0.631 | 1.364 |  | Gad1 | 0.718 | 0.884 |
| Crhr1 | 0.631 | 1.300 |  | Gabra2 | 0.728 | 0.969 |
| Dlg4 | 0.650 | 0.927 |  | Grin2b | 0.749 | 1.002 |
| Calb1 | 0.670 | 0.917 |  | Maob | 0.753 | 1.101 |
| Gad2 | 0.719 | 1.054 |  | Maoa | 0.781 | 0.895 |
| Grin2b | 0.727 | 0.959 |  | Drd1 | 0.793 | 1.077 |
| Fos | 0.749 | 1.182 |  | Gria1 | 0.795 | 0.934 |
| Igf1 | 0.759 | 0.930 |  | Ncan | 0.819 | 0.932 |
| Npas4 | 0.772 | 1.077 |  | Pvalb | 0.821 | 0.967 |
| Acan | 0.813 | 0.945 |  | Daglb | 0.832 | 0.932 |
| Rtn4r | 0.817 | 0.936 |  | Npas4 | 0.931 | 0.972 |
| Gad1 | 0.844 | 1.055 |  | Ntrk2 | 0.952 | 0.981 |
| Bdnf | 0.873 | 1.165 |  | Nrxn1 | 0.964 | 0.983 |
| Vip | 0.873 | 1.068 |  | Grin1 | 0.968 | 1.012 |
| Oxtr | 0.952 | 1.012 |  | Cck | 0.986 | 0.994 |
| Nlgn1 | 0.969 | 1.007 |  | Crhr1 | 0.988 | 0.995 |
| Grin2a | 0.978 | 0.992 |  | Igf2 | 1.000 | 0.863 |

**Table S2. Differentially expressed candidate genes in vulnerable subjects compared to the resilient group.**

| **Gene** | **p value** | **Fold change** |
| --- | --- | --- |
| **Ncan** | **0.005** | **0.241** |
| **Maoa** | **0.006** | **-0.259** |
| **Crh** | **0.011** | **1.440** |
| **Fos** | **0.011** | **-0.398** |
| **Rtn4r** | **0.021** | **0.626** |
| **Ngf** | **0.024** | **0.273** |
| **Gabra2** | **0.026** | **0.589** |
| **Daglb** | **0.026** | **0.239** |
| **Vip** | **0.026** | **0.088** |
| **Grin2b** | **0.029** | **0.284** |
| **Slc17a6** | **0.050** | **0.709** |
| Nfkb2 | 0.061 | -0.392 |
| Maob | 0.063 | 0.019 |
| Npy1r | 0.088 | 0.134 |
| Oxtr | 0.094 | -0.974 |
| Faah | 0.101 | 0.122 |
| Ngfr | 0.109 | -0.878 |
| Bdnf | 0.114 | -0.464 |
| Il1b | 0.125 | -0.765 |
| Adra2b | 0.143 | -0.793 |
| Htr1a | 0.148 | 0.052 |
| Hapln1 | 0.156 | 0.266 |
| Htr3a | 0.162 | 0.128 |
| Grm1 | 0.169 | 0.117 |
| Gria1 | 0.175 | 0.041 |
| Gabra1 | 0.180 | 0.212 |
| Npas4 | 0.213 | -0.126 |
| Adra1d | 0.215 | 0.102 |
| Grm5 | 0.220 | 0.066 |
| Lynx1 | 0.227 | 0.031 |
| Dagla | 0.231 | 0.037 |
| Arc | 0.246 | 0.096 |
| Crhr2 | 0.249 | 0.215 |
| Rtn4 | 0.251 | 0.034 |
| Tnr | 0.254 | 0.078 |
| Cck | 0.264 | -0.045 |
| Adra2a | 0.296 | 0.100 |
| Trpv1 | 0.307 | -0.086 |
| Adra1a | 0.345 | 0.020 |
| Drd5 | 0.354 | 0.097 |
| Htr2a | 0.357 | 0.033 |
| Comt | 0.363 | 0.034 |
| Npy2r | 0.381 | 0.059 |
| Slc17a7 | 0.400 | -0.017 |
| Nr3c1 | 0.420 | -0.026 |
| Cx3cl1 | 0.432 | 0.016 |
| Grin2a | 0.433 | 0.030 |
| Nfkb1 | 0.450 | 0.023 |
| Calb2 | 0.453 | 0.032 |
| Adcyap1r1 | 0.469 | 0.025 |
| Igf1 | 0.490 | -0.019 |
| Acan | 0.493 | -0.052 |
| Ntrk2 | 0.494 | -0.014 |
| Vcan | 0.515 | -0.022 |
| Fkbp4 | 0.516 | -0.011 |
| Nr3c2 | 0.541 | -0.041 |
| Fkbp5 | 0.542 | -0.014 |
| Igf2 | 0.549 | -0.260 |
| Grin1 | 0.581 | -0.018 |
| Il6 | 0.589 | 0.082 |
| Cnr1 | 0.602 | 0.011 |
| Htr2c | 0.636 | -2.413 |
| Dlg4 | 0.646 | 0.011 |
| Adrb2 | 0.684 | 0.016 |
| Pvalb | 0.685 | -0.013 |
| Tnf | 0.689 | -0.041 |
| Mgll | 0.693 | -0.005 |
| Nlgn2 | 0.707 | 0.009 |
| Drd1 | 0.740 | 0.027 |
| Bcan | 0.780 | -0.004 |
| Nlrp3 | 0.806 | 0.008 |
| Nrxn1 | 0.849 | 0.003 |
| Gria2 | 0.853 | 0.002 |
| Ncam1 | 0.868 | 0.002 |
| Adrb1 | 0.882 | -0.001 |
| Slc32a1 | 0.890 | -0.002 |
| Crhr1 | 0.911 | 0.001 |
| Calb1 | 0.918 | -0.001 |
| Lypd1 | 0.929 | 0.000 |
| Htr1b | 0.929 | -0.001 |
| Drd2 | 0.932 | 0.002 |
| Cx3cr1 | 0.936 | -0.001 |
| Nlgn1 | 0.937 | 0.000 |
| Nptx2 | 0.985 | 0.000 |
| Sst | 0.988 | 0.000 |
| Npy | 0.999 | 0.000 |
| Drd4 | N/A | N/A |
| Ifng | N/A | N/A |
| Ntrk1 | N/A | N/A |
| Slc6a2 | N/A | N/A |
| Slc6a3 | N/A | N/A |
| Slc6a4 | N/A | N/A |

**Table S3. Experimental details of cohorts included in the meta-analysis of freezing behavior across trauma exposure and fear recall sessions. Results of meta-analysis are shown in Supplementary Fig. S1.**

| **Experiment/Cohort#** | **N total (shocked groups)** | **Experiment** | **Related figure** |
| --- | --- | --- | --- |
| 1 | 35 | Pre-trauma affective-cognitive traits assessment | Fig.1 |
| 2 | 37 | ‘Simple’ operant learning assessment | Fig.2A |
| 3 | 31 | ‘Complex’ operant learning assessment | Fig.2D |
| 5A | 65 | Brain sampling for qPCR | Fig.4 |
| 5B | 31 | Brain sampling for Immunohistochemistry | Fig.4 |
| **5 cohorts** | **N=199 total** |  |  |

**Supplementary methods**

**Detailed materials and methods**

**Animals**

Subjects were adult (>10 weeks) male Long-Evans rats (Charles-River Laboratories, Italy), group-housed (4 rats/cage, 60 x 38 x 20 cm) and maintained at a controlled temperature of 22±1 °C and relative humidity of 50±10%. Water and laboratory food (Sniff, Germany) were provided *ad libitum*. Subjects were housed on a reverse 12-hour light/dark cycle (lights on at 8 p.m.). All experiments were carried out in accordance with the Directive of the European Parliament and the Council from 22 September 2010 (2010/63/EU) and were reviewed and approved by the Hungarian Government Office (PE/EA/874-5/2020), and the Animals Welfare Committee of the Institute of Experimental Medicine.

**Trauma exposure and fear generalization assessment: identification of resilient and vulnerable subpopulations**

Subjects were exposed to ten scrambled electric footshocks (2.4 mA; 10 ms pulses interrupted with 20 ms breaks; SuperTech Instruments, Hungary) delivered with 30 s inter-trial intervals (ITIs) during a single 7-min session that started with a 2.5 min habituation period. Non-shocked control animals were exposed to the same context without receiving footshocks. Contextual fear memory was briefly tested 28 days after trauma exposure for 5 min in the same context without delivering footshocks (i.e. CtxA): Plexiglas chamber of 40 x 40 x 40 cm dimensions equipped with a stainless steel electrical grid floor and striped sidewalls (Coulbourn Instruments, Holliston, MA, USA), bright white illumination (300 lux), apparatus cleaned with 20% ethanol between subjects. On the subsequent two days, subjects were exposed to an altered/novel context (CtxB1-2): oval-shaped Plexiglas chambers with plastic floor and solid white sidewalls, red light illumination (5 lux), cleaned with fruity-odor water, conducted by a different experimenter in a different experimental room; i.e. altering all visual, olfactory, and tactile components of the context) for 20 min to assess fear expression in a ‘safe’ context (i.e. fear generalization) (Fig. 1a and b). The index of fearful response was the time spent with freezing, a species-typical fear response in rodents, quantified with the activity analysis function of Ethovision XT15 software (Noldus, The Netherlands). Experimental populations/cohorts (n=32 or higher) were stratified into quartiles (25%) based on the freezing response exhibited in the two exposures to CtxB to define vulnerable and resilient subpopulations (i.e. high and low fear generalizers). Intermediate quartiles (middle 50%) were not used in our study, except when correlation analysis was conducted on the whole population.

**Behavioral testing**

All behavioral tests were conducted during the early dark phase, video recorded and analyzed by Ethovision XT15 software except tests in operant chambers or the startle system, where behavioral data were directly collected from automated apparatus, and the social recognition test where behavior was analyzed by an event-based recorder (https://solomon-coder.software.informer.com/). Boxes were cleaned with water and wiped dry between subjects if not stated otherwise.

**Anxiety tests: light-dark box, elevated plus-maze, and open field**

The Light-Dark Box consisted of a transparent well-lit compartment (50 x 50 x 40 cm, 300 lux) joined to a dark/covered compartment (30 x 50 x 40 cm). Subjects were placed in the light compartment and were allowed to explore for 10 min. The Elevated Plus-Maze was a black arena with 42 x 12 cm arms (35 cm wall height) at 70 cm height and illuminated by dim red light (5 lux). Subjects were placed in the center facing the closed arm and were allowed to explore for 5 min. The Open Field arena was a black non-transparent plastic box (79 x 54 x 35 cm, center defined as 40 x 27 cm) with low-intensity illumination (100 lux). Subjects were placed in the corner and were allowed to explore for 10 minutes. In all tests, time spent in the aversive zone, the number of entries, and latency to enter were considered as indexes of anxiety. We also combined these variables into composite z-scores for a more reliable index of anxiety. The total distance moved was the index of locomotor activity.

**Startle response assessment**

Startle reactivity was measured in ventilated, sound-attenuating startle chambers (33 x 33x 48 cm; SR-LAB Startle Response System, San Diego Instruments, USA) containing a Plexiglas cylinder (length: 25 cm, d=12 cm). A speaker 24 cm above the cylinder provided background noise (65 dB) and the startle stimuli (20 ms pulses of 80, 90, 100, 110, and 120 dB intensities). The startle session started with a 5-min acclimation period followed by five 120 dB pulses to reach more stable responses. Next, we presented four of each pulse intensities in a pseudo-random order (average 15 s ITIs) to assess the average startle response for each intensity.

**Predator odor avoidance test**

Innate fear response was assessed by exposing subjects to a synthetic analogue of a fox anogenital odor product, 2-methyl-2-thiazoline (2MT; M83406, Sigma Aldrich) presented in a Plexiglas arena (43 × 27 × 19 cm, 50ul 2MT in a plastic cap at the corner) under bright illumination (300 lux). Subjects were placed in the opposite corner and were allowed to explore for 10 min. Entries and time spent near the 2MT source (7 × 11 cm defined zone), and time spent with freezing were indexes of innate fear. We also calculated a composite z-score similar to anxiety tests.

**Y-maze**

Y-maze was made of three interconnected Plexiglas arms (45 x 17 x 30 cm with 120° angles). Subjects were placed in a dedicated start arm and were allowed to explore for 5 min. Number and sequence of turns made between arms were quantified to calculate the ratio of spontaneous alternation as the index of working memory.

**Social recognition test**

The procedure was adapted from Engelmann and his colleagues (Engelmann et al., 2011). Briefly, subjects were habituated in plastic testing cages (60 x 40 x 50 cm, dim illumination) for 2 h before presenting a same-sex juvenile rat (30-35 days old) to interact for 4 min (sampling phase). After a 30min memory retention period, they were introduced to the same familiar juvenile rat and a novel same-sex juvenile rat for 4 min (social recognition phase). Time spent investigating each juvenile was scored manually by a trained observer. Time preference for the novel juvenile rat was considered as social recognition index.

**Y-place context recognition test**

The apparatus was made of transparent Plexiglas arms (each arm: 45 x 17 x 30 cm with 80^o^ angle between choice arms). Choice arms were contextualized by placing distinct objects outside along the walls. Subjects were placed in the start arm and allowed to explore for 10 min with one choice arm closed (sampling phase). After a 30-min memory retention phase spent in their home cage, subjects were re-placed into the start arm and were allowed to explore the arena with two choice arms for 5 min (choice phase). Time preference of the novel arm was the index of place recognition.

**Morris water maze**

The water maze was a black circular pool of 180 cm in diameter and 60 cm in height filled with 21 °C (room temperature) tap water, which was made opaque by white tempera paint. A white platform of 12 cm diameter was placed in the northwest quadrant and submerged 1.5 cm below the surface. The water maze was surrounded by different visual objects around the pool serving as extra-maze visual cues (under dim illumination, 70 lux). Animals were placed in the pool at one of the five releasing positions for four daily trials for four days. The order of releasing positions was randomized daily but kept constant between subjects. All trials lasted until the animal found the hidden platform (max. 90 s). If the animal did not find the platform during the trial, it was guided to the platform. All subjects were left to stay on the platform for an additional 15 s, then transferred to the next releasing point (start of the next trial). Average daily latency to find the platform (i.e. escape latency) was an index of learning performance. After completing the trials, animals were gently and thoroughly dried with cotton towels before returning to their home cage.

**T-maze**

The apparatus was made of three Plexiglas arms (45 x 17 x 30 cm) interconnected in a T-shape. Distinct intra-maze cues contextualized the two choice arms: plastic inserts on the walls (dotted vs. striped) and floors (LEGO plate vs. smooth plastic) at the entry zones. The test started with repeated habituation to the maze (5 min daily for 3 days) followed by a training phase (6 trials daily with 30 s it is for 13 days) when one of the choice arms was rewarded by a cornflake (at the end of the arm). The trial ended if the rat turned into either choice arm (and consumed the reward in case of baited arm) or 120 s elapsed without a turn into the arms. Animals were trained to reach the criterion of 5 correct trials (choice of rewarded arm) out of 6. The maze was cleaned with 20% ethanol and wiped dry between trials. In the test phase, the start arm was re-arranged (i.e., moving to the opposite side, from south to north position), while choice arms remained in the same position. Rats were given four test trials to follow either habitual response strategy (turn to the same direction as during training trials) or contextual response strategy (turn based on intra-maze cue, i.e. in opposite direction) in their choices. The frequency of these two strategies was the index of habitual and contextual strategy preference.

**Simple operant learning and Go/No-Go tasks**

These tasks were performed in daily training sessions using automated operant chambers equipped with nose poke holes with infrared sensors and LED lights, and a food pellet receptacle in a central position between the nose poke holes (Med Associates, St. Albans, VT, USA). All chambers were located in ventilated sound-attenuating cubicles and controlled by MED-PC IV software. Four days before testing, subjects were put on a restrictive diet (weights maintained at 80-90% of *ad libitum* weight). The task started with two simple operant learning phases (30 min sessions) when a LED light cue indicated the rewarded hole. Cue length was 30 s and 10 s during phases 1 and 2, respectively. Rewards were 45mg dustless precision sucrose pellets (BioServ, USA) gained by nose poking into the correct (cued) hole. Correct or incorrect nose pokes terminated the trials, or an omission was registered if no poking occurred. Trials were separated by 5 s intertrial intervals (ITIs) when the house light was turned off to distinguish the active trials from ITIs. The criterion for entering the next phase was >80% accuracy (i.e. the ratio of correct responses) on two consecutive days (applied for all tasks described below). Phase 2 operant task was followed by the Go/No-Go task (40 min sessions), where subjects had to inhibit their response when the No-Go signal (5 s long acoustic cue, 50% of trials in pseudorandom order) was co-presented with the light cue (Go signal for 5 s) to earn a reward. A response during a No-Go signal (false alarm) terminated the trial without reward. During the Go/No-Go task, each trial started with a 9-24 s variable pre-cue period when nose pokes during the last 3 s of this period were considered premature responses, which re-started the trial. Registered variables were the total number of responses, percentage of correct and incorrect trials (accuracy), number of premature responses, number of omissions, and days to reach the criterion to qualify for the next phase.

**Complex operant learning, strategy set-shifting, and 5-choice serial reaction time test (5-CSRTT)**

The same operant boxes were equipped with five nose poke holes on the opposite wall to the food receptacle. Subjects had to learn a more complex operant task (30 min sessions) where the cue light (30 s, illuminating one out of the five nose poke holes randomly across trials) was an irrelevant stimulus since the location of the nose poke hole (either 2nd or 4th hole, randomly assigned and counterbalanced within the sample) indicated the rewarded hole. The reward was delivered for correct pokes during the cue period or subsequent 10 s limited hold period. House light indicated active trial that was turned off during 5 s ITIs. The operant task was followed by a strategy set-shifting task (30 min sessions) where subjects had to switch from a location-based response strategy to a visual cue strategy as the light cue became the relevant stimulus predicting the rewarded nose poke hole position. Finally, subjects entered a 5-choice serial reaction time task (5-CSRTT, based on (Bari et al., 2008)). During this task, subjects had to poke during cue periods (with subsequent 10 s hold-in period) with progressively shortening lengths (from 20 s to 1.25 s) during a 30-min session. Failure to respond during cue or subsequent limited hold periods (omitted trial) or response to non-cued holes (incorrect trial) were registered.

**Post-trauma operant training**

One week after the fear generalization assessment in CtxB, vulnerable and resilient subpopulations were sorted into 'trained' and 'yoked control' groups (counterbalanced for their average freezing level). ‘Trained’ groups were exposed to the same complex operant learning task with five poke holes as described above, whereas ‘yoked controls’ were exposed to operant chambers with random rewarding schedule, i.e. collected the same amount of rewards (median pellet numbers collected by the trained groups on the previous day) that were not dependent on their behavioral performance, but given by a random algorithm. After completing the 80% accuracy criterion, subjects were put back on *ad libitum* food access for a week before testing contextual fear recall (5 min in CtxA) and fear generalization (20 min in CtxB) to measure the impact of operant training on fear generalization and extinction. Noteworthy, the complex operant paradigm was chosen for training based on (1) its highest prediction power for fear generalization (potentially with over-lapping networks with fear expression), (2) its cognitively challenging nature recruiting these prefrontal networks, and (3) its feasibility characteristics (within 4 weeks that is comparable with post-trauma incubation time before recall testing).

**Gene expression analysis by quantitative polymerase chain reaction (qRT-PCR)**

Immediately, after fear generalization testing in CtxB, rats were decapitated to collect brain samples: 1 mm thick coronal sections were cut (~Bregma 3.70 mm to 2.70 mm; (Paxinos and Watson, 2007)) for the medial prefrontal cortex samples, which was dissected between the ventral point of the forceps minor (~DV 5.40) and the dorsal border of the Cg1 (i.e. including the IL, PrL, and Cg1, although with notable area dominance towards PrL). Samples were snap-frozen and kept at -80°C until gene expression analysis. Total RNA was isolated and treated to remove genomic DNA contamination using RNeasy Lipid Tissue Mini Kit (Qiagen, The Netherlands) according to the manufacturer's protocol. RNA quality and quantity were measured (Agilent 2100 Bioanalyzer, Agilent Technologies, USA or Qubit RNA IQ Assay Kit and Qubit RNA BR Assay Kit, Invitrogen, USA). 1 µg total RNA of each sample was reverse transcribed (High capacity cDNA reverse transcription kit, Thermo Fisher Scientific, USA), and cDNA concentrations were determined (Qubit ssDNA assay kit, Invitrogen, USA). cDNA samples were diluted and combined with TaqMan Gene Expression Master Mix (1 ng/µl final concentration) to create a PCR reaction mix. 92 or 44 candidate genes and four housekeeping genes were analyzed using custom 384-well TaqMan Gene Expression Array Cards (Applied Biosystems, USA). qRT-PCR was performed (ViiA 7 Real-Time PCR System, Applied Biosystems, USA), and data was collected using the QuantStudio software (Applied Biosystems, USA). Gene expression levels were normalized to the two most stable endogenous control genes across reaction samples (*Actb* and *Gapdh*; geNorm method, Vandesompele, 2002, Genome Biology), and their relative quantity was calculated by the 2^-ddct^ method (Lival, 2001, Methods). Six genes were excluded from the analysis due to low expression levels (Ct numbers >35).

**Immunohistochemical labeling**

90 min after fear generalization assessment in CtxB, subjects were deeply anesthetized (ketamine-xylazine-pipolphen: 200-40-25 mg/kg) and transcardially perfused with cold 0.1 M phosphate-buffered saline (PBS) followed by 4% paraformaldehyde (PFA) in PBS solution. Brains were post-fixed for 3 h, then transferred to 30% sucrose/PBS solution before sectioning 30 µm thick coronal sections using a sliding microtome. Samples were stored at -20 °C in a cryoprotectant solution before immunohistochemical labeling.

First, we labeled c-Fos as an activity marker to map the neuronal activity of the fear-regulating network during fear generalization (CtxB). We labeled medial prefrontal cortex (3 coronal sections at +3.70, +3.20, and +2.70 mm to Bregma), paraventricular thalamic nucleus (3 coronal sections at -2.30, -2.80, and -3.14 mm to Bregma), central amygdala (2 coronal sections at -2.30, and -2.80 mm to Bregma) and basolateral nuclei of the amygdala (3 coronal sections at -2.30, -2.80, and -3.14 mm to Bregma), and CA1-CA3 and dentate gyrus subregions of the dorsal hippocampus (4 coronal sections at -2.30, -2.56, -2.80, and -3.20 mm to Bregma), and ventral hippocampus (6 coronal sections from -4.80 to -5.80 mm to Bregma); (Paxinos and Watson, 2007). Second, we co-labeled c-Fos with cell-type specific markers (somatostatin - SST, parvalbumin - PV, calretinin - CR, vasoactive intestinal peptide - VIP), and monoaminergic fibers (serotonin transporter-SERT, tyrosine hydroxylase-TH and dopamine beta hydroxylase-DBH) in mPFC (Bregma 3.70-2.70 mm) to characterize network functioning using multiple fluorescent immunolabeling. Briefly, free-floating sections were washed in Tris buffer saline (TBS), and incubated in a blocking solution containing 10% normal donkey serum (NDS; Jackson ImmunoResearch, UK) with 0.3% TritonX-100 in TBS for 1 h. Then slices were incubated in primary antibody solution at 4 ^o^C for 72 h (diluted in TBS with 5% NDS and 0.1% TritonX-100). The following primary antibodies were used: guinea pig anti-c-Fos (1:3000, Synaptic systems, #226-004), mouse anti-NeuN (1:2000, Millipore, #MAB377), rabbit anti-VIP (1:500, Immunostar, #20077), mouse anti-calretinin (1:2000, Swant, #6B3), and rabbit anti-parvalbumin (1:5000, Swant, #PV 27), rabbit anti-somatostatin-14 (1:10000, Peninsula Laboratories International, Inc., #T-4103), anti-TH (1:2000, Millipore, #AB152), mouse anti-DBH (1:2000, Millipore, #MAB308) and guinea pig anti-SERT (1:2000, Synaptic Systems, #340004). Incubation was followed by TBS washes and 2 h long incubation in secondary antibody solution (1:500, with Alexa Fluor 647, Cy3, or Alexa Fluor 488 conjugates, and Hoechst staining in 1:2000 dilution) at room temperature. Finally, slices were washed in TBS, mounted on glass slides, and coverslipped using Mowiol4-88 (Merck).

**Microscopy and image analysis**

Slides were imaged using a Pannoramic Digital Slide Scanner (Pannoramic MIDI II; 3DHISTECH, Hungary). An experimenter blind to the experimental groups defined the section planes and the anatomical structures based on reference atlas (Paxinos and Watson, 2007) and manually annotated the mPFC subregions using the CaseViewer 2.4 software (3DHISTECH, Hungary). We also annotated L2/3, L5, and L6 layers based on previous reports (Van Eden and Uylings, 1985; Van De Werd et al., 2010; Nagy-Pal et al., 2023), using general cell density (NeuN) and parvalbumin distribution (Supplementary Fig. S4B). The total c-Fos signal was counted by using a custom-written ImageJ script. The interneuron marker VIP, CR, PV, and SST positive cells and their co-localization with c-Fos signal were manually quantified using the ImageJ software with the Cell counter plugin. Immunopositive signals (i.e. cell numbers) were counted bilaterally (2-3 sections 360 µm apart) in annotated areas in the above-mentioned regions of interest. Signal densities (cell/mm^2^) were calculated and averaged across sections and bilateral areas.

TH, DBH, and SERT immunostaining were imaged using a Nikon C2 Confocal Microscope with a 20x objective (Plan Apo VC NA=0.75 WD=1mm FOV=645.12um, Nikon). Three focal planes were captured from the PrL area as a z-stack series using the Large area module. Images were converted to maximum-intensity projection images for fiber density analysis, and further processed by background subtraction in ImageJ. Plot Profile function over a standardized area over the cortical layers provided an average value of the intensity of every pixel column (aligned parallel with layers) in our selected area, which was used as an index of fiber density related to every layer.

**Knockdown of *Crh* expression using shRNA vectors**

Adeno-associated virus (AAV) vectors expressing small hairpin RNAs (shRNA) targeting *Crh* or scrambled base sequence (AAV5-EGFP-Scramble_shRNA, #VB010000-0023jze, scr) as control were purchased from VectorBuilder (Chicago, USA). First, the most effective construct (#VB900052-5912cnc: cnc) was selected from three AAV5-EGFP-rCrh constructs (#VB900052-5919rbk: rbk; #VB900052-5925bpn: bpn; 10e13 GC/ml titers) by injecting each into the mPFC (n=3 animals per group) and quantifying mPFC *Crh* gene expression two and four weeks later using TaqMan qRT-PCR using *Crh* primer Rn01462137_m1 (Thermo Fisher), normalized to *Gapdh* expression (fig. S6).

For our behavioral experiment, cnc construct was selected and injected bilaterally into mPFC at the level of PrL and IL cortices (2 x 0.5 µl volume/hemisphere: using AP 2.7 mm, ML 0.5 mm, DV -3.6 and -4.0 mm coordinates from Bregma; Paxinos, 1994) through a glass pipette (tip diameter: 20–30 μm) at a rate of 200 nl/min by using a Nanoject II precision microinjector pump (Drummond, Broomall, PA, USA) under ketamine-xylazine-pipolphen anesthesia (intraperitoneally, 41,6mg/kg; 8,3mg/kg, 4,16mg/kg, respectively) using stereotaxic equipment (David Kopf Instruments, Tujunga, CA, USA). The pipette was left in place for an additional 5 min after injection to ensure diffusion before slow retraction. After the surgeries, rats received buprenorphine injection (0.1 mg/kg) subcutaneously as analgesic treatment. Surgery was conducted two days after trauma exposure (to avoid interference with the acquisition, and to manipulate the fear incubation period). Four weeks after the virus injection, fear recall tests were conducted as described above. 90 min after fear generalization assessment in CtxB, subjects were anesthetized and transcardially perfused to verify virus infection sites using immunolabeling against the green fluorescent protein (EGFP) imaging (expressed by virus vectors). Only subjects with infection sites limited to mPFC were included in our analysis.

**Machine learning classification**

To assess feature importance, we utilized Random Forest Classifiers implemented in the scikit-learn library in Python. For model optimization we performed hyperparameter tuning using a grid search, cross-validating the model across five iterations. This ensured robust evaluation and selection of the best-performing parameters. The average feature importances were derived from 30 independent predictions.

**Statistical analysis**

Data are presented as mean ± standard error of the mean. Statistical analysis was performed using GraphPad Prism (GraphPad, USA), Statistica (Tibco, USA), and R statistical environment. Data were analyzed using Student’s t-test, one- or two-way ANOVA with Tukey's post hoc test, and repeated measures ANOVA. When the test prerequisites of ANOVA were not fulfilled, we used the non-parametric Mann-Whitney U test. Pearson test was used to assess correlations, where data from animals of intermediate quartiles were also included. Distributions and their modality were analyzed by the multimode R package (Muller 1991). Statistical significance was set at *p*<0.05 in all cases.

**References**

Bari A, Dalley JW, Robbins TW (2008) The application of the 5-choice serial reaction time task for the assessment of visual attentional processes and impulse control in rats. Nat Protoc 3:759-767.

Engelmann M, Hadicke J, Noack J (2011) Testing declarative memory in laboratory rats and mice using the nonconditioned social discrimination procedure. Nat Protoc 6:1152-1162.

Nagy-Pal P, Veres JM, Fekete Z, Karlocai MR, Weisz F, Barabas B, Reeb Z, Hajos N (2023) Structural Organization of Perisomatic Inhibition in the Mouse Medial Prefrontal Cortex. J Neurosci 43:6972-6987.

Paxinos G, Watson C (2007) The Rat Brain in Stereotaxic Coordinates, 6th Edition: Academic Press, San Diego, CA.

Van De Werd HJ, Rajkowska G, Evers P, Uylings HB (2010) Cytoarchitectonic and chemoarchitectonic characterization of the prefrontal cortical areas in the mouse. Brain Struct Funct 214:339-353.

Van Eden CG, Uylings HB (1985) Cytoarchitectonic development of the prefrontal cortex in the rat. J Comp Neurol 241:253-267.
